# TRAM-LAG1-CLN8 family proteins are acyltransferases regulating phospholipid composition

**DOI:** 10.1126/sciadv.adr3723

**Published:** 2025-02-19

**Authors:** Pradeep K. Sheokand, Andrew M. James, Benjamin Jenkins, Pawel K. Lysyganicz, Denis Lacabanne, Martin S. King, Edmund R. S. Kunji, Symeon Siniossoglou, Albert Koulman, Michael P. Murphy, Kasparas Petkevicius

**Affiliations:** ^1^ MRC Mitochondrial Biology Unit, University of Cambridge, Cambridge Biomedical Campus, Cambridge, UK.; ^2^ Metabolic Research Laboratories, Institute of Metabolic Science, University of Cambridge, Cambridge Biomedical Campus, Cambridge, UK.; ^3^ Cambridge Institute for Medical Research, University of Cambridge, Cambridge Biomedical Campus, Cambridge, UK.; ^4^ Department of Medicine, University of Cambridge, Cambridge Biomedical Campus, Cambridge, UK.

## Abstract

The diversity of cellular phospholipids, crucial for membrane homeostasis and function, arises from enzymatic remodeling of their fatty acyl chains. In this work, we reveal that poorly understood TRAM-LAG1-CLN8 domain (TLCD)–containing proteins are phospholipid remodeling enzymes. We demonstrate that TLCD1 is an evolutionarily conserved lysophosphatidylethanolamine acyltransferase, which regulates cellular phospholipid composition and generates previously undescribed fatty acid and thiamine (vitamin B1) esters as its secondary products. Furthermore, we establish that human TLCD protein CLN8, mutations of which cause fatal neurodegenerative Batten disease, is a lysophosphatidylglycerol acyltransferase. We show that CLN8 catalyzes the essential step in the biosynthesis of bis(monoacylglycero)phosphate, a phospholipid critical for lysosome function. Our study unveils a family of acyltransferases integral to cellular membrane phospholipid homeostasis and human disease.

## Teaser

TRAM–LAG1–CLN8 family proteins function as enzymes that remodel cellular phospholipids and expand the membrane lipid diversity.

## INTRODUCTION

The ability to dynamically remodel membrane phospholipid acyl chains allows cells to adapt to changing extracellular environments and alter the morphology and function of their intracellular organelles (*1*, *2*). Cellular phospholipid remodeling is predominantly mediated by the enzymes of the 1-acylglycerol-3-phosphate *O*-acyltransferase (AGPAT) and membrane-bound *O*-acyltransferase (MBOAT) families (*3*). These enzymes catalyze the esterification of various fatty acyl-coenzyme A (CoA) species onto the glycerol moiety of lysophospholipids, thus creating a diverse array of membrane phospholipid species (*3*).

**Fig. 1.**
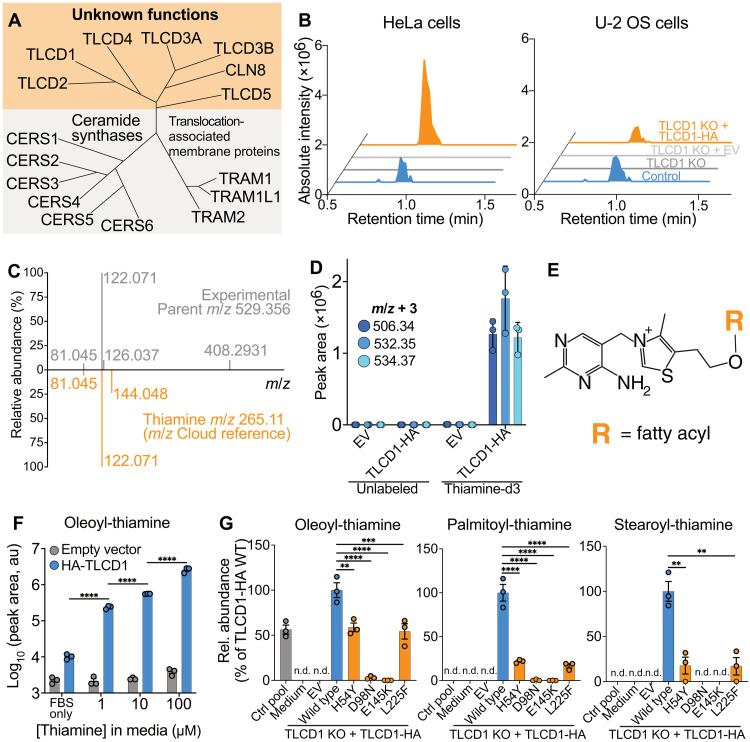
Discovery and validation of acyl-thiamine species in cultured cells. (**A**) Simplified human TLCD protein phylogeny. (**B**) Representative aligned 529.35 *m*/*z* ion chromatograms from HeLa and U-2OS control and TLCD1 KO clones, transfected with either empty vector (EV), or TLCD1-HA plasmids. (**C**) Mirror plots comparing MS/MS spectra from 529.35 *m*/*z* experimental fragmentation and from thiamine fragmentation at *m*/*z* Cloud database. (**D**) Abundance of m+3 species (indicative of thiamine-d3 labeling) in TLCD1 KO HeLa pool, transfected with EV or TLCD1-HA, and treated with either thiamine-d3 or normal culture medium. (**E**) Chemical structure of acyl-thiamine species. (**F**) Abundance of oleoyl-thiamine in HeLa TLCD1 KO pool transfected with TLCD1-HA, cultured in varying amounts of thiamine as indicated (*n* = 3). au, arbitrary units. (**G**) Relative acyl-thiamine levels in control and TLCD1 KO HeLa cells, transfected with EV or plasmids encoding TLCD1-HA variants, presented as percentages of the WT TLCD1-HA transfection baseline (*n* = 3). n.d., not detected; **P < 0.01, ***P < 0.001, and ****P < 0.0001 using one-way analysis of variance (ANOVA) with Sidak’s post hoc test [(F) and (G)]. Data were obtained in [(B), (C), and (G)] an untargeted, and in [(D) and (F)] a targeted manner.

Phosphatidylethanolamine (PE) is the second most abundant mammalian membrane phospholipid, enriched in mitochondria and the inner layer of the plasma membrane (*4*). Previously, we observed that poorly understood proteins TLCD1 and TLCD2 regulate PE composition in mouse liver and promote the progression of non-alcoholic fatty liver disease (*5*). In addition, the *Caenorhabditis elegans* TLCD1 homolog Membrane Fluidity Homeostasis-1 (FLD-1) was identified as a key regulator of membrane lipid composition in worms subjected to saturated fatty acid (SFA)–induced lipotoxic conditions (*1*). Both TLCD1 and FLD-1 belong to the TLCD family of proteins, which contain 16 members in the human genome (Fig. 1A and fig. S1A) (*6*). Six human TLCD proteins have a well-defined function as ceramide synthases (CERSs), catalyzing the incorporation of fatty acyl-coenzyme As (CoAs) onto sphingoid bases (*7*). However, the biochemical functions of TLCD1, FLD-1, and the remaining TLCD proteins—specifically the mechanism through which they regulate membrane lipid composition—remains unknown.

## RESULTS

### TLCD1 generates fatty acyl-thiamine esters in cultured cells

To elucidate the mechanism of action of TLCD1, we engineered TLCD1 knockout (KO) HeLa and U-2OS cells and compared their lipidomes to isogenic wild-type (WT) controls (fig. S2, A to B). Consistent with our previous findings in mouse liver (*5*), TLCD1 KO HeLa and U-2OS cells both exhibited altered PE fatty acyl compositions compared to their respective controls (fig. S3, A to D). This alteration was readily evident in CRISPR-edited cell pools and more pronounced in populations derived from single clones (fig. S3, A to D). Although our current lipidomics methodology could not discern the composition of individual PE acyl chains, the bulk species data, coupled with our earlier observations in TLCD1 KO mouse liver (*5*), suggested a decrease in monounsaturated fatty acid–containing PE species in TLCD1 KO HeLa and U-2OS cells. Furthermore, we analyzed mitochondrial PE composition by genetically tagging the mitochondria in HeLa TLCD1 KO and control pools and performing lipidomics on immunoprecipitated mitochondria. Similar to the mitochondria isolated from mouse liver (*5*), HeLa TLCD1 KO mitochondria exhibited altered PE composition compared to controls (fig. S4, A to C). Overall, our findings indicate that TLCD1 regulates cellular and mitochondrial PE composition in both mouse liver and human cultured cell lines through an unidentified mechanism.

During lipidomics analysis, we identified an unknown lipid species present in controls but absent in TLCD1 KO pools and clones (Fig. 1B and fig. S5A). This species, designated as 529.35 due to its main mass/charge ratio (*m*/*z*) signal, was a singly charged protonated ion that did not match any known molecular entities in metabolite databases. The abundance of 529.35 could be restored by transfecting TLCD1 KO cells with hemagglutinin (HA)–tagged TLCD1 (Fig. 1B and fig. S5A). In addition, we observed two other chemically related unknown species—*m*/*z* 503.34 and 531.37—which were only present in cells transfected with TLCD1-HA (fig. S5, A to F). Mass spectrometry (MS)/MS fragmentation analysis of 529.35 revealed shared fragment ions with thiamine (vitamin B1) (Fig. 1C). On the basis of the *m*/*z* differences between the newly identified species, we hypothesized that they could be different fatty acids conjugated to thiamine. To investigate this, TLCD1 KO cells were cultured and transfected with either an empty vector or TLCD1-HA in the presence of stably labeled fatty acids or thiamine. All species could be labeled with d3-thiamine (Fig. 1D). U-^13^C_16_-palmitic acid labeled 503.34; U-^13^C_18_-stearic acid labeled 531.37, and U-^13^C_18_-oleic acid labeled 529.35 (fig. S5G). Collectively, our results indicate that 503.34 represents thiamine palmitate, 529.35 represents thiamine oleate, and 531.37 represents thiamine stearate. The fragmentation analysis of stable isotopically labeled acyl-thiamine species indicated that fatty acyl groups are attached to the hydroxyl group of thiamine via an ester bond, which is also the site of phosphorylation of thiamine pyrophosphokinase (Fig. 1E and fig. S6, A to B).

In our cell culture experiments, we used Dulbecco’s modified Eagle’s medium (DMEM) that contains approximately 10 μM thiamine. Modifying thiamine concentration in cell culture media proportionally altered cellular acyl-thiamine levels (Fig. 1F and fig. S5H). In addition, we engineered TLCD1-HA variants with single amino acid substitutions analogous to the loss-of-function FLD-1 mutations identified in the prior *C. elegans* screen (fig. S1B) (*1*). Apart from the T152P mutant, which could not be detected in transient transfection experiments in HeLa cells, the other TLCD1 mutants were expressed at levels similar to the WT and retained their Golgi localization (fig. S7, A to B). Notably, the expression of the H54Y and L225F mutants in TLCD1 KO HeLa cells resulted in a substantial reduction in acyl-thiamine species compared to cells expressing the WT TLCD1-HA (Fig. 1G). Moreover, TLCD1 KO cells expressing D98N and E145K mutants in highly conserved TLCD1 residues exhibited a near-complete absence of acyl-thiamine species (Fig. 1G and fig. S1B). These results collectively suggest that the newly found cellular lipids—fatty acyl-thiamines—are products of the enzymatic action of TLCD1.

### TLCD1 is a lysophosphatidylethanolamine acyltransferase

**Fig. 2.**
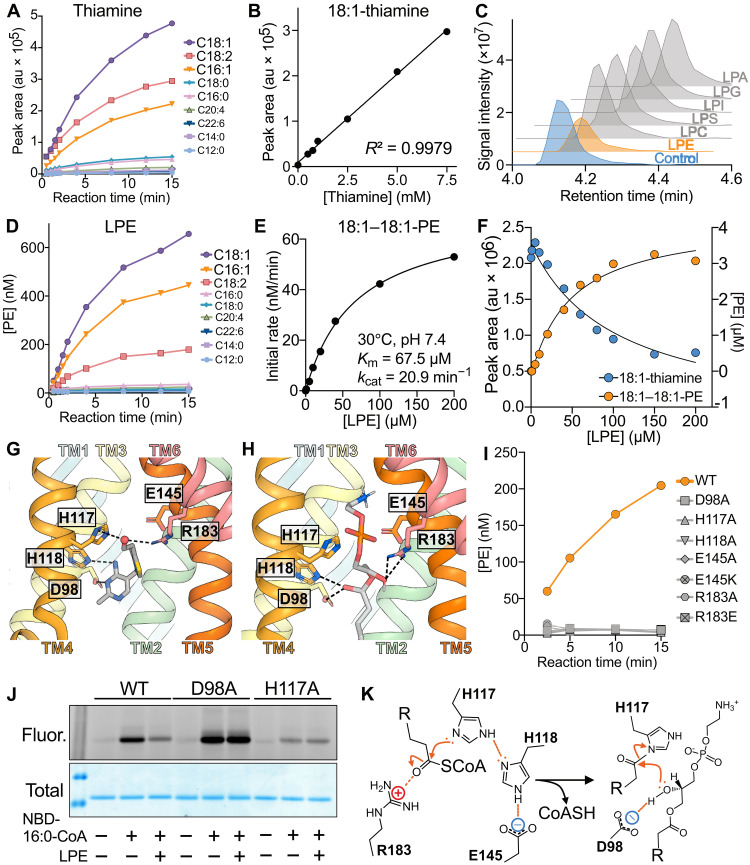
Characterization of TLCD1 as an LPE acyltransferase operating in a double displacement kinetic mechanism. (**A**) Time-dependent quantification of indicated acyl-thiamine species in reactions containing purified TLCD1, equimolar acyl-CoA mix, and thiamine. au, arbitrary units. (**B**) Linear regression between thiamine concentration and the oleoyl-thiamine synthesis rates in *V*_0_ assays using purified TLCD1 with oleoyl-CoA and thiamine. (**C**) Aligned chromatograms showing targeted oleoyl-thiamine detection in assays using purified TLCD1 with oleoyl-CoA and 500 μM thiamine, with the addition of 500 μM specified lysophospholipids. LPC, lysophosphatidylcholine; LPE, lysophosphatidylethanolamine; LPS, lysophosphatidylserine; LPI, lysophospatidylinositol; LPG, lysophosphatidylglycerol; LPA, lysophosphatidic acid. (**D**) Time-dependent quantification of indicated acyl-18:1-PE species in reactions containing purified TLCD1, equimolar acyl-CoA mix, and 18:1 LPE. (**E**) Michaelis-Menten plot of *V*_0_ assays with purified TLCD1, oleoyl-CoA, and increasing 18:1-LPE concentrations. (**F**) Concurrent measurement of 18:1-thiamine (left y-axis) and 18:1–18:1-PE (right *y* axis) in assays with purified TLCD1, oleoyl-CoA, 500 μM thiamine, and varying 18:1-LPE concentrations. Molecular docking of (**G**) thiamine and (**H**) 18:1-LPE to the AlphaFold2-predicted structure of TLCD1, zoomed in on the putative active site containing conserved residues of TLCD family. (**I**) Time-dependent quantification of 18:1–18:1 PE in assays using purified TLCD1 variants with oleoyl-CoA and 18:1-LPE. (**J**) In-gel NBD fluorescence (top) and total protein Coomassie staining (bottom) of SDS-PAGE of purified TLCD1 variants, incubated with NBD-16:0-CoA alone or together with 18:1-LPE for 5 min at 30°C as indicated. (**K**) Proposed TLCD1 reaction mechanism. All data shown are representative of at least two independent protein purifications. All MS/MS data were obtained in a targeted manner.

We sought to understand the enzymatic activity of TLCD1 by purifying it from human cells. Despite TLCD1 forming SDS-resistant dimers and oligomers in denaturing gel electrophoresis, size exclusion chromatography (SEC) indicated a single native TLCD1 population (fig. S8, A to C). In two-dimensional cryo–electron microscopy classifications, TLCD1 was observed as a monomer embedded within detergent micelles (fig. S9, A to B). Incubating TLCD1 with thiamine and an equimolar mix of fatty acyl-CoAs and monitoring the production of acyl-thiamines by liquid chromatography tandem MS (LC-MS/MS) revealed a time-dependent increase in fatty acyl-thiamine species, indicating enzymatic activity that favored mono- and diunsaturated fatty acyl-CoA substrates (Fig. 2A). We also observed the formation of fluorescent nitrobenzoxadiazole (NBD)–palmitoyl–thiamine with NBD-palmitoyl-CoA substrate (fig. S10A). The reaction rate with saturating concentrations of oleoyl-CoA and thiamine substrates was directly proportional to the TLCD1 concentration (fig. S10B). Kinetic analysis with saturating oleoyl-CoA concentration across a range of thiamine concentrations showed a linear increase in the initial reaction rate (*V*_o_) even at millimolar thiamine levels (Fig. 2B), suggesting that thiamine, due to its nanomolar in vivo presence (*8*), might not be the optimal substrate of TLCD1.

Considering TLCD1 KO models exhibit altered phospholipid composition, we investigated the interaction of various lysophospholipids with thiamine during TLCD1 catalysis. Lysophosphatidylethanolamine (LPE) suppressed oleoyl-thiamine formation, whereas other lysophospholipids had no effect (Fig. 2C). Unexpectedly, despite having no homology to lysophospholipid acyltransferases in AGPAT and MBOAT families (*3*), TLCD1 produced a fluorescent compound with similar mobility to a PE standard on thin-layer chromatography (TLC) using NBD-palmitoyl-CoA and LPE as substrates (fig. S10C). Confirming this by LC-MS/MS, TLCD1 successfully generated PE from LPE and an equimolar fatty acyl-CoA mix, favoring monounsaturated fatty acyl-CoA substrates (Fig. 2D). The kinetic characterization of TLCD1 showed that its affinity and catalytic efficiency for both oleoyl-CoA and LPE substrates were comparable to known phospholipid remodeling enzymes in AGPAT and MBOAT families (Fig. 2E and fig. S10E) (*9*, *10*). In addition, TLCD1 could transfer acyl groups to both *sn-*1-acyl or *sn-*2-acyl LPE (fig. S10D). Collectively, these findings elucidate the role of TLCD1 as an acyltransferase that preferentially incorporates monounsaturated fatty acids into PE, aligning with the altered PE composition observed in TLCD1 KO mouse and cell models (*5*).

To assess whether the detergent used for purifying and assaying TLCD1 affected its substrate preference and selectivity, we compared the activities of TLCD1 purified and assayed in lauryl maltose neopentyl glycol (LMNG), which had been used in our experiments so far, with its activity in other detergents, namely, decyl maltose neopentyl glycol (DMNG), dodecyl-β-d-maltoside (DDM), and glyco-diosgenin (GDN). TLCD1 was successfully solubilized and purified to a similar extent with all four detergents (fig. S11A). TLCD1 exhibited similar activity with LPE as a substrate in LMNG and GDN, while no activity was observed in DMNG or DDM (Fig. S11B). TLCD1 showed comparable activity for thiamine as a substrate in both LMNG and DDM but was inactive for thiamine in DMNG and GDN (fig. S11C). Moreover, TLCD1 activity could not be saturated at supraphysiological thiamine concentrations in either LMNG or DDM (fig. S11C). Overall, LMNG was the only detergent in which TLCD1 exhibited activity for both substrates, leading us to continue using it for subsequent experiments.

To investigate whether LPE and thiamine share the same binding site on TLCD1, we incubated TLCD1, thiamine, and oleoyl-CoA with increasing concentrations of LPE, and simultaneously monitored both acyl-thiamine and PE products. We observed an inverse relationship between acyl-thiamine and PE production, indicating that thiamine and LPE compete for the same active site (Fig. 2F). Molecular docking simulations using AlphaFold2-predicted TLCD1 structure demonstrated that both thiamine and LPE bind similarly within the central cavity, engaging conserved TLCD1 residues recently implicated as the active site in the TLCD1 paralog CERS6 (Fig. 2, G to H, and figs. S1B and S9, C to D) (*11*). Mutagenesis of these residues resulted in loss of TLCD1 activity toward both substrates, underscoring their functional importance (Fig. 2I and fig. S12, A to D). As a homologous residue to TLCD1 H117 in CERS6 was recently shown to form a stable acyl-enzyme reaction intermediate (*11*), we investigated if TLCD1 also operates by forming an analogous intermediate. Given that TLCD1 could use NBD-palmitoyl-CoA as a substrate, we addressed this by monitoring the in-gel fluorescence of purified TLCD1, preincubated with NBD-palmitoyl-CoA alone or with LPE, and subjected to denaturing gel electrophoresis. We observed fluorescent NBD-palmitoyl-TLCD1 intermediate, which was diminished in the presence of LPE (Fig. 2J). All TLCD1 variants tested, except for D98A, showed an impaired ability to form this intermediate (fig. S12E). Intriguingly, the D98A mutant accumulated the fluorescent intermediate, which was not deesterified in the presence of LPE, indicating that it was “locked” in an intermediate state (Fig. 2J). This observation suggests that the D98 residue is required for the transfer of the acyl group from H117 to LPE or thiamine (Fig. 2K). Together, our results demonstrate that TLCD1 shares a conserved active site with its paralog CERS6 and follows a similar double-displacement (ping-pong) kinetic mechanism, proceeding through an acyl-enzyme intermediate (Fig. 2K) (*11*).

### TLCD1 enzymatic function is conserved in yeast and worm

**Fig. 3.**
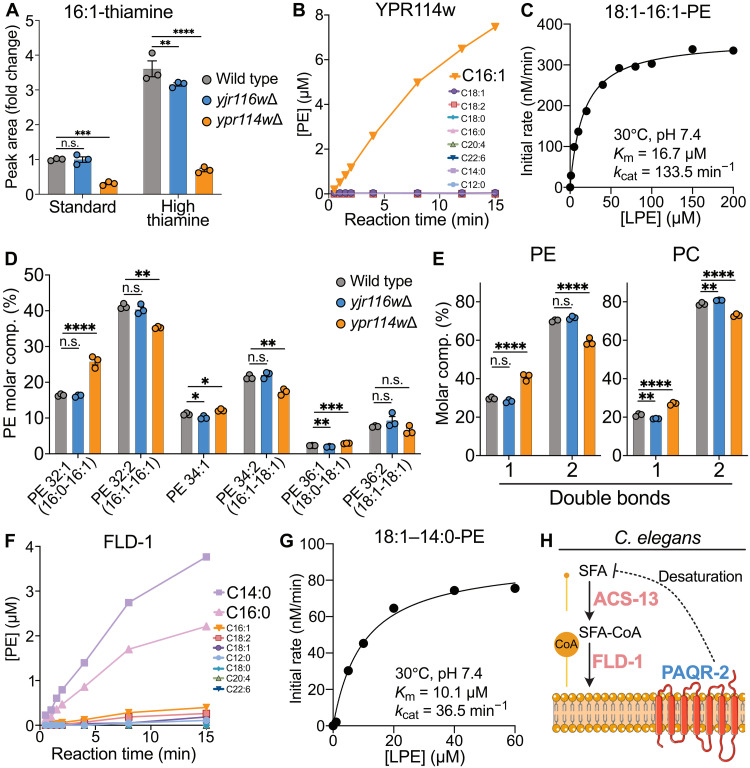
Characterization and functional importance of TLCD1 evolutionary homologs YPR114w and FLD-1. (**A**) Levels of 16:1-thiamine species (normalized to WT control condition) in WT, *ypr114w*Δ, or *yjr116w*Δ *S. cerevisiae* strains, cultured in regular medium or supplemented with 100 μM thiamine (*n* = 3). (**B**) Time-dependent quantification of indicated acyl-18:1-PE species in reactions containing purified YPR114w, equimolar acyl-CoA mix, and 18:1-LPE. (**C**) Michaelis-Menten plot of *V*_0_ assays with purified YPR114w, palmitoleoyl-CoA and increasing 18:1-LPE concentrations. (**D**) PE molar composition and (**E**) relative abundance of double bonds in PE and PC in WT, *ypr114w*Δ, or *yjr116w*Δ *S. cerevisiae* strains (*n* = 3). (**F**) Time-dependent quantification of indicated acyl-18:1-PE species in reactions containing purified FLD-1, equimolar acyl-CoA mix, and 18:1-LPE. (**G**) Michaelis-Menten plot of *V*_0_ assays with purified FLD-1, myristoyl-CoA, and increasing 18:1-LPE concentrations. (**H**) Proposed *C. elegans* pathway*—*ACS-13 and FLD-1 incorporate saturated fatty acids (SFAs) into membranes, where membrane fluidity sensor PAQR-2 feeds back by increasing fatty acid desaturation to maintain membrane homeostasis. The in vitro assay results are representative of at least two independent protein purifications. n.s., not significant; *P < 0.01, **P < 0.01, ***P < 0.001, and ****P < 0.0001 using [(A) and (E)] two-way ANOVA and (D) one-way ANOVA with Dunnett’s post hoc tests. Data were obtained in (D) and (E) an untargeted and in (A) to (C) and (F) to (G) a targeted manner.

To explore whether TLCD1 enzymatic activity is conserved in yeast, we generated two *Saccharomyces cerevisiae* mutants harboring the deletion of *TLCD1* homolog genes *ypr114w* and *yjr116w* (fig. S1A). These mutants have been independently generated previously and found not to have growth defects (*12*), consistent with our observations (fig. S13A). We cultured these strains under standard conditions (with 1.3 μM thiamine) and exposed them to a high thiamine concentration of 100 μM before analyzing acyl-thiamine species. The sole acyl-thiamine species detectable in *S. cerevisiae*—palmitoleic acid (C16:1)-thiamine ester—was elevated under high thiamine and reduced in *ypr114w*Δ, but not in *yjr116w*Δ cells compared to WT controls across both conditions (Fig. 3A). This indicated that YPR114w could be an enzyme catalyzing a reaction similar to TLCD1. Purified YPR114w demonstrated the ability to transfer acyl-CoAs to thiamine and LPE, with a remarkable specificity for palmitoleoyl-CoA (Fig. 3B and fig. S14, A to D). YPR114w exhibited a higher turnover number and a lower *K*_m_ (Michaelis constant) for both substrates compared to TLCD1 (Fig. 3C and fig. S14E). To determine whether YPR114w regulates phospholipid composition in *S. cerevisiae*, we performed lipidomic analysis of *ypr114w*Δ and *yjr116w*Δ cells. Although total phosphatidylcholine (PC) and PE levels were unaltered in mutants (fig. S13B), *ypr114w*Δ cells had reduced palmitoleic acid–containing PE species compared to WT controls (Fig. 3D). This reduction was offset by an increase in saturated fatty acid–containing PEs (Fig. 3D), a pattern that is remarkably similar to our observations in TLCD1 KO mouse liver and human cells (fig. S3, A to D) (*5*). Given that yeast primarily produces PC from PE (*13*), the *ypr114w* deletion also caused similar shifts in the acyl chain composition of PC species, ultimately increasing the saturation levels of both primary membrane phospholipids (Fig. 3E and fig. S13C). On the basis of our collective findings, we designate YPR114w as ALE2 (acyltransferase for lysophosphatidylethanolamine 2), recognizing it as the second identified yeast LPE acyltransferase. Notably, although ALE2 has no sequence homology to the previously characterized MBOAT family member ALE1, both enzymes share the same function but differ in their acyl-CoA substrate specificity (*14*).

To investigate whether FLD-1, the *C. elegans* homolog of TLCD1, may also function as an acyltransferase, we purified codon-optimized FLD-1 from yeast to assess its enzymatic activity in vitro (fig. S15, A to B). FLD-1 efficiently catalyzed acyl-CoA transfer to both LPE and thiamine, with a strong preference toward saturated fatty acyl-CoA substrates, namely, myristoyl-CoA (C14:0) and palmitoyl-CoA (C16:0) (Fig. 3, F to G, and fig. S15, C to E). *Fld-1*, together with *acs-13* (encoding acyl-CoA synthase), were previously identified as genetic suppressors of the phenotypes of *paqr-2* mutant worms, which have impaired fatty acid desaturation and exhibit lethality in the presence of elevated dietary SFAs (*1*, *15*). The enzymatic function of FLD-1 found here offers a clear molecular mechanism for the previously observed genetic interactions: In the absence of PAQR-2, the activation of SFAs by ACS-13 and incorporation of SFA-CoA to PE by FLD-1 drive lethality in SFA-rich conditions in worms, where PE is the most abundant membrane phospholipid species (Fig. 3H) (*1*). Together, the enzymatic function and the role in regulating membrane lipid composition of TLCD proteins are conserved across yeast, worm, mouse, and human species.

### CLN8 is a lysophosphatidylglycerol acyltransferase in BMP synthesis pathway

**Fig. 4.**
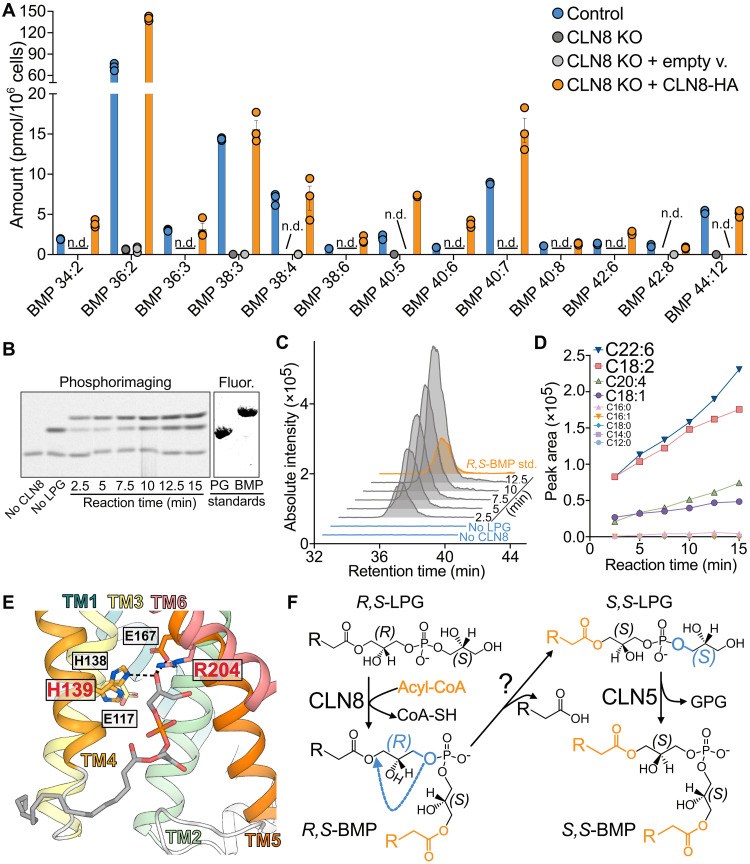
Characterization of TLCD1 paralog CLN8 as a LPG acyltransferase. (**A**) Levels of indicated BMP species measured in HeLa control and CLN8 KO pools, transfected with either empty vector (EV), or CLN8-HA plasmids (*n* = 3). (**B**) Phosphorimaging of time-course assays with purified CLN8, ^14^C-oleoyl-CoA, and 18:1-LPG, separated by TLC. A fluorescence scan of primulin-stained 18:1–18:1-PG and *R*,*S*-18:1–18:1-BMP standards separated on the same plate is presented alongside. (**C**) Aligned chromatograms showing the BMP-specific *m*/*z* 775.5 to 339.3 MS/MS transition measured in timecourse assays with CLN8, oleoyl-CoA, and 18:1-LPG. *R*,*S*-18:1–18:1-BMP standard is shown in orange. (**D**) Time-dependent quantification of indicated BMP species in reactions containing purified CLN8, equimolar acyl-CoA mix, and 18:1-LPG. (**E**) Molecular docking of 18:1-LPG to the AlphaFold2-predicted structure of CLN8, zoomed in on the putative active site containing conserved residues of TLCD family. Residues mutated in Batten disease are indicated in red. (**F**) Proposed pathway of cellular BMP biosynthesis, adapted from an earlier proposal by Heravi and Waite (*19*). The acyl groups added by CLN8 are shown in orange, while the glycerol reorientation from *R* to *S* conformation is highlighted in blue. GPG, glycerophosphoglycerol byproduct from the CLN5 reaction. The in vitro assay results are representative of two independent protein purifications. Data were obtained in (A) an untargeted and in (C) to (D) a targeted manner.

Last, we investigated whether other human TLCD family proteins function as lysophospholipid acyltransferases. We chose to investigate TLCD1 paralog CLN8 (also known as TLCD6) (Fig. 1A and fig. S1A) because mutations in *CLN8* gene cause Batten disease, a fatal neurodegenerative lysosomal storage disorder occurring in children (*16*). Similar to TLCD1, CLN8 localizes to the endoplasmic reticulum (ER)–Golgi network and was previously described as a cargo receptor mediating the trafficking of soluble lysosomal proteins (*17*, *18*). To explore the role of CLN8 in phospholipid metabolism, we generated HeLa and U-2OS CLN8 KO CRISPR-edited pools and compared their lipidomes to the WT controls (fig. S16, A and B). Strikingly, we found a near-complete absence of lysosomal phospholipid bis(monoacylglycero)phosphate (BMP) species in both HeLa and U-2OS CLN8 KO pools (Fig. 4A and fig. S16, C and D). Transfection with CLN8-HA completely restored BMP levels in HeLa CLN8 KO pool and only restored some of the depleted BMP species in U-2OS CLN8 KO pool (Fig. 4A and fig. S16, C and D). Notably, U-2OS showed a distinctive profile of BMP species compared to HeLa cells, particularly enriched in BMP lipids containing docosahexaenoic acid (DHA; 22:6) (fig. S16D). Collectively, our lipidomics findings indicated that CLN8 may be involved in BMP biosynthesis, and we opted to investigate it further in vitro.

BMP is distinctive among other phospholipids; it features a phosphate group linked to the *sn-1* positions of the glycerol moyeties rather than the typical *sn-3* position (*19*). The previously proposed model for BMP biosynthesis suggests that lysosomal BMP, in its mature *S*,*S*-stereoconfiguration (with both glycerol moieties containing chiral carbons in the *S* conformation), is synthesized in cells through a two-stage process. Initially, an unidentified enzyme catalyzes the synthesis of BMP in an *R*,*S*-stereoconfiguration using *R*,*S*-lysophosphatidylglycerol (LPG) as a precursor (*19*). Subsequently, this *R*,*S*-BMP intermediate undergoes two additional enzymatic steps to be converted into *S*,*S*-BMP (*19*). We hypothesized that CLN8 may perform the initial *R*,*S*-BMP synthesis by acylating the headgroup of LPG. Because LPG can be acylated to form either phosphatidylglycerol (PG) or BMP, we aimed to distinguish between these reaction products in assays using purified CLN8 (fig. S17, A to C). Our results showed that purified CLN8 preferentially synthesized BMP in a time-dependent manner during an assay with 18:1-LPG and [1-^14^C]-oleoyl-CoA, as determined by the migration of the radiolabeled CLN8 reaction product relative to the *R*,*S*-dioleoyl-BMP standard in TLC (Fig. 4B). This finding was confirmed by monitoring the BMP-specific monoacylglycerol fragment transition by LC-MS/MS in time-course assays with purified CLN8, 18:1-LPG, and unlabeled oleoyl-CoA (Fig. 4C and fig. S17D). When assayed with an equimolar acyl-CoA mix and 18:1-LPG, CLN8 showed a preference for polyunsaturated linoleoyl-CoA (18:2) and DHA-CoA substrates (Fig. 4D). Molecular docking of 18:1-LPG to the AlphaFold2-predicted structure of CLN8 revealed the conserved TLCD family residues, which are essential for TLCD1 activity, surrounding the LPG headgroup (Fig. 4E and figs. S1B and S17E). Notably, mutations in conserved histidine and arginine residues have been frequently observed in CLN8 in patients with Batten disease (H139Y, R204C, and R204L mutations) (*20*–*23*). Overall, we found that CLN8 is an acyl-CoA–dependent acyltransferase catalyzing the essential step of *R*,*S*-BMP intermediate formation in the lysosomal *S*,*S*-BMP synthesis pathway.

## DISCUSSION

Here, we show that TLCD family proteins, which were poorly characterized, function as acyltransferases in membrane phospholipid remodeling. Unlike the MBOAT and AGPAT enzymes (*24*, *25*), TLCD enzymes have a smaller active site and cannot simultaneously accommodate both substrates. Instead, our data indicate that they operate in a ping-pong mechanism analogous to CERS enzymes within the same family (*11*), beginning with an acyl-enzyme intermediate formation, and followed by the acylation of the lysophospholipid substrate. It is plausible that such a mechanism makes TLCD enzymes more prone to react with other substrates containing similar chemical groups to those in lysophospholipids, as exemplified here with thiamine and LPE, both of which contain positively charged moieties near hydroxyl groups that undergo acylation. As TLCD1 has been previously implicated in the development of non-alcoholic steatohepatitis and liver cancer (*5*, *26*), it may be of interest to develop TLCD1 inhibitors based on the structure of thiamine and explore their therapeutic potential.

Moreover, this study is the first to identify acyl-thiamines and demonstrate their presence in human and yeast cells cultured in media with supraphysiological levels of thiamine. Although our data suggest that thiamine may not be the optimal substrate for TLCD1, as the reaction could not be saturated even at very high thiamine concentrations in vitro, there are inherent limitations to our in vitro assays. Specifically, TLCD1 embedded in biological membranes rather than in detergent micelles may allow different access for thiamine to its active site or facilitate better diffusion of the acyl-thiamine product into the surrounding membrane, potentially altering substrate affinity and reaction kinetics. Furthermore, different cell types may have other enzymes capable of acylating thiamine with higher affinity. Because of the absence of a chemical standard for acyl-thiamine, we could not quantify its concentration in cells and compare it to that of free thiamine or its biological derivatives, such as thiamine pyrophosphate. Future research should aim to validate the presence of acyl-thiamines in mouse and human tissues and accurately quantify absolute acyl-thiamine levels under different physiological or pathological conditions using synthetic acyl-thiamine as an internal standard. It is intriguing to consider that acyl-thiamines might be involved in specific biological scenarios, such as sequestering thiamine within lipid environments, which warrants investigation.

Last, we show that the TLCD family protein CLN8 functions as an acyltransferase, catalyzing the headgroup acylation of LPG to produce *R*,*S*-BMP. Our results indicate that this reaction is a critical step in lysosomal *S*,*S*-BMP biosynthesis, as CLN8-deficient cells lack BMP species. Another Batten disease–related protein CLN5 was recently identified as a lysosomal enzyme that synthesizes BMP using two LPG molecules (*27*). Yet, without other enzymes to catalyze the *R*,*S*-LPG stereoisomerization, CLN5 could only form *R*,*S*-BMP and not mature lysosomal *S*,*S*-BMP (*19*). We propose that CLN8 catalyzes the acylation of *R*,*S*-LPG in the ER/Golgi to produce the *R*,*S*-BMP intermediate. This molecule then undergoes a rearrangement, whereby the phosphoryl ester migrates from the *sn*-3 to the *sn*-1 position, releasing the *sn*-1 linked acyl chain (Fig. 4F) (*28*). This migration, catalyzed by an unidentified enzyme, results in the formation of the *S*,*S*-LPG intermediate, which is trafficked to the lysosome, where CLN5 uses it to generate *S*,*S*-BMP (Fig. 4F). This proposed pathway aligns with prior evidence that fatty acyl-CoA synthesis is necessary for BMP biosynthesis (*29*) and that BMP formation involves the removal of both acyl groups while retaining both glycerol moieties from the precursor PG during its conversion (*28*, *30*). Our finding underscores lysosomal phospholipid dysregulation as a common pathological mechanism in Batten disease variants and highlights the importance of the BMP synthesis pathway.

## MATERIALS AND METHODS

### Materials

The detailed list of antibodies used in this study is provided in table S1. The detailed list of chemicals and reagents used in this study is provided in table S2.

#### Molecular biology

All gene synthesis in this paper was performed by GenScript. All plasmids were validated by Sanger sequencing (performed by Source Genomics), and plasmid list is provided as table S3. DH5α competent cells (18265017, Thermo Fisher Scientific) were used for cloning. Phusion Flash High-Fidelity PCR Master Mix (F548L, Thermo Fisher Scientific) was used for all polymerase chain reaction (PCR) reactions. All mutagenesis was performed using a classic two PCR method, and resulting PCR products were inserted into the same vectors as WT controls.

To generate plasmids for transient TLCD1 expression studies, the following synthesized sequence was cloned into pcDNA3.1(+)-C-HA vector: atgccccgactgctgcaccccgccctgccgctgctcctgggcgccacgct-gaccttccgggcgctccggcgcgcgctctgtcgcctgcccctacccgtgcacgtgcgcgccga-ccccctgcgcacctggcgctggcacaacctgctcgtctccttcgctcactccattgtgtcggggatctgggcactgctgtgtgtatggcagactcctgacatgttagtggagattgagacggcgtggtcactttctggctatttgctcgtttgcttctctgcggggtatttcatccacgatacggtggacatcgtggctagcggacagacgcgagcctcttgggaataccttgtccatcacgtcatggccatgggtgccttcttctccggcatcttttggagcagctttgtcggtgggggtgtcttaacactactggtggaagtcagcaacatcttcctcaccattcgcatgatgatgaaaatcagtaatgcccaggatcatctcctctaccgggttaacaagtatgtgaacctggtcatgtactttctcttccgcctggcc-cctcaggcctacctcacccatttcttcttgcgttatgtgaaccagaggaccctgggcacctt-cctgctgggtatcctgctcatgctggacgtgatgatcataatctacttttcccgcctcctccgctctgacttctgccctgagcatgtccccaagaagcaacacaaagacaagttcttgactgagggctcatccggtggtggtgggtccggcgggggcggttcaagtgga.

To make plasmids for inducible Flp-In T-REx 293 cell line generation, the following TLCD1 sequence (containing different C-terminal tag) was cloned into pcDNA5/FRT/TO vector: atgccccgactgctgcaccccgccctgccgctgctcctgggcgccacgctgaccttccgggcgctccggcgcgcgctctgtcgcctgcccctacccgtgcacgtgcgcgccgaccccctgcgcacctggcgctggcacaacctgctcgtctccttcgctcactccattgtgtcggggatctgggcactgctgtgtgtatggcagactcctgacatgttagtggagattgagacggcgtggtcactttctggctatttgctcgtttgcttctctgcggggtatttcatccacgatacggtggacatcgtggctagcggacagacgcgagcctcttgggaataccttgtccatcacgtcatggccatgggtgccttcttctccggcatcttttggagcagctttgtcggtgggggtgtcttaacactactggtggaagtcagcaacatcttcctcaccattcgcatgatgatgaaaatcagtaatgcccaggatcatctcctctaccgggttaacaagtatgtgaacctggtcatgtactttctcttccgcctggcccctcaggcctacctcacccatttcttcttgcgttatgtgaaccagaggaccctgggcaccttcctgctgggtatcctgctcatgctggacgtgatgatcataatctacttttcccgcctcctccgctctgacttctgccctgagcatgtccccaagaagcaacacaaagacaagttcttgactgagctcgagggaaccggtggagctggcgattacaaggacgacgatgacaagggcggagccgctggatggagccacccccagttcgagaagtgatga.

To generate a plasmid for transient CLN8 expression studies (including purification), the following synthesized sequence was cloned into pcDNA3.1(+)-C-HA vector: atgaatcctgcgagcgatgggggcacatcag-agagcatttttgacctggactatgcatcctgggggatccgctccacgctgatggtcgctggctttgtcttctacttgggcgtctttgtggtctgccaccagctgtcctcttccctgaatgccacttaccgttctttggtggccagagagaaggtcttctgggacctggcggccacgcgtgcagtctttggtgttcagagcacagccgcaggcctgtgggctctgctgggggaccctgtgctgcatgccgacaaggcgcgtggccagcagaactggtgctggtttcacatcacgacagcaacgggattcttttgctttgaaaatgttgcagtccacctgtccaacttgatcttccggacatttgacttgtttctggttatccaccatctctttgcctttcttgggtttcttggctgcttggtcaatctccaagctggccactatctagctatgaccacgttgctcctggagatgagcacgccctttacctgcgtttcctggatgctcttaaaggcgggctggtccgagtctctgttttggaagctcaaccagtggctgatgattcacatgtttcactgccgcatggttctaacctaccacatgtggtgggtgtgtttctggcactgggacggcctggtcagcagcctgtatctgcctcatttgacactgttccttgtcggactggctctgcttacgctaatcattaatccatattggacccataagaagactcagcagcttctcaatccggtggactggaacttcgcacagccagaagccaagagcaggccagaaggcaacgggcagctgctgcggaagaagaggcca.

To generate a plasmid for YPR114w expression and purification from yeast, the following synthesized sequence was cloned into pYES2/CT vector: atggatgttttattgtcgcttcctcaaccggaattatttaagaccacg-gtgattccgttcttggcaaatcgcaatataatcaagtcggaagcgattctctccaacttgcactcaattttttatgttgctatattctaccatatttggtttctttttggcaaatggatcttattcccacatttggttaaatggaaattggactatgaccaaaaacataacgtcaagaaagatgagaagacaacttcggaacgtcaagctcaacattacaaaaagaagtacacttctttgatcaatcaaagttcagtccacttaatatccctactgcaaagcatagtggtcctgtactactcattgaagttcttgcttgatccaaaagcctcggccgagccctaccaaacctcacactcccgagtgtttacagaaaatcgagacactcaagtcatctgtatttttgctattggttatttcgtctgggatatctatatttccaccatgtattctactttccccttcgttgtgcacggaataatctccaccgtcgtgttttgcatcggattgaaaccgtacatccaatattacgccccagtgttcttgatgttcgaactttccaatccctccttgaactttagatggttcggtatcaaatttctaccccagaaaagcaaattctgctctctactgctgctgttgaacaatttgacgctcatggtcgtcttctttgccgctagaatcgcctgggggtggtttcaaattggaaaactatgttacgacttttaccaggtgcgcaatgaacctggtttcttagtctttgacaccattgttatccttgcaggcaatttcgtcctggacatcttgaatgttatttggttttctaccatggtgtctgtggctgcaaaggtcttgaagaagggggagtctgtagacaaagtcactaagaatgaacaagatgcagcaattgaaggtaggacatctgaagatcatcatcaccatcaccatcatcattgataatag.

To generate a plasmid for FLD-1 expression and purification from yeast, the following synthesized sequence (codon optimized for yeast expression) was cloned into pYES2/CT vector: atgagacaattggccgaattgttaaccgatttattaggcccagttccaactatgttcttgtgggttattgtctctttcgctttctttagagctttgcaattcatcgttagatggtacttgtttggtaagtggacctggccaaattttaacttcttcgacatcagaaaccgtatccgtagaagaagaagaggtggtcaagaagctgaaaacaccgaaaacccacctgaaaatgaagctgaagccggtgaacaagttgaacaagaaccagaaccagatagccgtgacttgtccgctatcccaccaaacaagaaatggagaatctctaacgaatgtgttagtctattccattccgtcatttccggtttatgggctgcttacgctttgttatactacaagcaattggtccaagatttggttaactacagatgtgatgttgctattaacttggtcttgatgtctgctggttacttgttccacgacttagttgacttgttggtcaacgaacaatccgctagaatcattgaattgttgttccaccacgttgttgttttatctgccttcgctgttaccatgttcttcaacagattcttgggtgttgtcgttttcggtttgttgatggaactgaactctatcttcttgcactccagatctttgttgaacttgtacggtgttgacaagaagtctccatctttcagaattattgctttgttaaacatggttactttgtttgccttcagattgtgtgtctcagcatatttggtctacttcgttgtcgtctccattccagacttggaatggtacgtttccatcatcaacggtttggtcatcgcttctttggcttccaccaacactgttttgacttacagactacttgccgctgacggtctgttgggttctcgtagaaccaggagaactccagctgctaccgctgaaactcaagtcggtgacgtcgaatctggtccattgagaacccaagttgaagatgaagaccaccacactatcggtgttcaaacaattcacggtaccactgaagatgccactcaaaccgtcgatgctgccattgaaggtagaacttctgaagaccatcaccaccaccaccatcatcactgataatag.

### Human cell culture

HeLa [European Collection of Authenticated Cell Cultures (ECACC) 93021013] cell line was obtained from Culture Collections, UK Health Security Agency (catalog no. 93021013). U-2 OS cell line was obtained from Culture Collections, UK Health Security Agency (catalog no. 92022711). Flp-In T-REx 293 cell line was purchased from Thermo Fisher Scientific (catalog no. R78007). All cell lines were maintained in DMEM (11965084, Thermo Fisher Scientific) supplemented with 10% fetal bovine serum (FBS; Sigma-Aldrich) at 37°C and 5% CO_2_. Cells were passaged at 90% confluency using TrypLE Express Enzyme (12605010, Thermo Fisher Scientific). Cells were routinely tested for mycoplasma contamination.

For transient transfection experiments, cells grown in their standard culture medium were transfected using FuGENE HD Transfection Reagent (E2311, Promega) according to the manufacturer’s protocol. For a single well of a six-well plate, 20 μl of Opti-MEM (31985062, Thermo Fisher Scientific), 0.4 μg of plasmid DNA, and 1.2 μl of FuGENE HD were used. Medium was refreshed the next day and cells were analyzed 48 to 72 hours post-transfection.

To collect samples for lipid measurements, confluent cells in six-well plates were swiftly washed twice with 3 ml of ice-cold PBS on ice and then scraped into 500 μl of ice-cold PBS on ice using inverted 200 μl pipette tip. The resulting cell suspension was transferred into prechilled 2-ml plastic vials used for lipid extraction (3469-11, Thermo Fisher Scientific) and centrifuged at 500*g*, 4°C for 5 min. The supernatant was aspirated, and the cell pellet was snap-frozen and stored at −70°C. One well of a six-well plate yielded one replicate in analysis.

To prepare thiamine-deficient culture media, DMEM High Glucose with l-glutamine and without thiamine (D9803-02, US Biological) was used. The powder was reconstituted in water containing sodium bicarbonate as per the manufacturer’s specifications. Thiamine concentrations were adjusted by adding thiamine hydrochloride (catalog number 102658221, Sigma-Aldrich) to the media to achieve the desired final concentrations. For labeling with deuterated thiamine, thiamine-d3 hydrochloride was added into the thiamine-free medium to a final concentration of 10 μM, and the cells were incubated for 24 hours.

For fatty acid labeling, U-^13^C_16_ palmitic acid, U-^13^C_18_ stearic acid, and U-^13^C_16_ oleic acids were initially solubilized in ethanol to create a 100 mM stock solution. This solution was then added dropwise to a complete DMEM medium, which had been supplemented with 1% bovine serum albumin, to achieve a final fatty acid concentration of 50 μM. The mixtures were sonicated until the solutions appeared clear. Following sonication, the fatty acid–enriched media was used to incubate cells for a period of 24 hours.

### Human KO cell line generation

The generation of HeLa and U-2 OS KO cell lines was accomplished by transfecting the cells with recombinant complexes comprising Cas9 and synthetic guide RNA. For this process, the cells were plated in their standard culture medium within six-well plates and subjected to transfection the subsequent day, at which point they reached an approximate confluence of 30%. The transfection utilized Lipofectamine CRISPRMAX Cas9 Transfection Reagent (CMAX00001, Thermo Fisher Scientific), TrueCut Cas9 Protein v2 (A36497, Thermo Fisher Scientific), and Invitrogen TrueGuide Synthetic gRNAs (custom synthesized by Thermo Fisher Scientific), all in adherence to the manufacturer’s guidelines. The following guideRNA pairs were used for KO generation: TLCD1 (TAGTGGAGATTGAGACGGCG and CCACGATGTCCACCGTATCG) and CLN8 (CGTTGCTCCTGGAGATGAGC and GACATTTGACTTGTTTCTGG). The KO wells received a transfection mixture containing 6250 ng of Cas9 nuclease, along with 600 ng each of targeting gRNA1 and gRNA2 to establish KO cell pools. Concurrently, control wells were transfected with 6250 ng of Cas9 nuclease and 1200 ng of nontargeting gRNA (A35526, Thermo Fisher Scientific), resulting in control cell pools. The culture medium was refreshed 24 hours post-transfection.

After another 24 hours following the medium refreshment, the contents from each well were transferred to 6-cm plates and concurrently sorted as single cells into 96-well plates—two plates for each well—using an Influx Cell Sorter (Becton Dickinson). The sorted cells were allowed to grow, while the remaining unsorted control and KO cell pools were subsequently expanded from the 6-cm plates to multiple 15-cm plates before being preserved by freezing in multiple aliquots. An aliquot from this preservation was then revived in culture, and the successful gene deletion was confirmed through PCR. For subsequent experiments, the frozen aliquots were used, ensuring they underwent no more than four passages.

Two weeks after the single-cell sorting, the control and KO clones were duplicated into two identical sets of 96-well plates. One set from each clone type was subjected to deletion validation via PCR and Sanger sequencing (performed by Source Genomics). Clones with confirmed deletions (or confirmed WT sequence for control clones) were then expanded further and frozen for future experiments.

### Cell line generation for mitochondrial immunoprecipitation

HeLa control or TLCD1 KO 3XMyc-EGFP-OMP25 and 3XHA-EGFP-OMP25 stable cell lines were generated according to the published protocol (*31*). To generate retrovirus, HEK293 cells in 10-cm plates were transfected with 3XMyc-EGFP-OMP25 or 3XHA-EGFP-OMP25 (5 μg), together with pL_VSVG (1.5 μg), pJK3 (3 μg) and pCMV_TAT_HIV (3.5 μg) plasmids, and 39 μl of FuGENE HD. Cell medium was refreshed 24 hours post-transfection, and retrovirus was allowed to produce for a further of 24 hours. Cell culture supernatants containing retrovirus were then collected, passed through 0.45-μm filter, aliquoted, snap-frozen, and stored at −70°C.

To generate stable cell lines, HeLa control or TLCD1 KO pools cultured in six-well plates were infected with retrovirus [200 μl of retrovirus-containing supernatant and polybrene (10 μg/ml) per well]. Medium was refreshed 24 hours later, and cells were allowed to grow for another 48 hours. Cells were then passaged into 6-cm plates and selected with blasticidin (10 μg/ml) for 2 weeks (passaging when reaching full confluency) before sorting low green fluorescent protein (GFP)–expressing populations (bottom 15% GFP intensity) using an Influx Cell Sorter (Becton Dickinson). The mitochondrial localization of GFP signal was validated by confocal microscopy.

### Human Flp-In T-REx 293 inducible cell line generation

Flp-In T-REx 293 cells cultured in standard culture medium in six-well plates were transfected at 25% confluency. The mixture of 20 μl of Opti-MEM, 0.36 μg of pOG44 plasmid, 0.04 μg of pcDNA5/FRT/TO plasmid with TLCD1 WT or mutant insert, and 1.2 μl of FuGENE HD was used for transfection. Medium was refreshed 24 hours later, and cells were cultured for a further 48 hours. Cells were then passaged into 6-cm plates and selected using standard culture medium containing hygromycin B (50 μg/ml) for 2 weeks, passaging when reaching confluency. Hygromycin B–resistant pools were then expanded, aliquoted, and frozen. An aliquot from this preservation was then revived in culture and tested for protein expression by dose response (at 48 hours) and time-course (at established optimal dose) induction with tetracycline.

### Yeast mutant generation and culture

Yeast *ypr114w*Δ, *yjr116w*Δ, and WT control cells were all in the BY4741 background (Open Biosystems). ﻿Gene deletion of *yjr116w* by chromosomal integration of the HphNT1 module was generated by one-step PCR-based method using as a template the pFA6a-based knock out cassette (*32*). Gene deletion of *ypr114w* by chromosomal integration of the KanMX4 module was generated by one-step PCR-based method using as a template the genomic DNA of the strain from the gene deletion collection (Open Biosystems). Deletions were confirmed by PCR.

Yeast cells were grown in synthetic medium (SC) containing 2% glucose, 0.17% yeast nitrogen base (catalog no. 233520, Difco, BD, Franklin Lakes, NJ), 0.5% ammonium sulfate, and amino acid drop-out [leucine (60 mg/liter), adenine (55 mg/liter), uracil (55 mg/liter), tyrosine (55 mg/liter), arginine (20 mg/liter), histidine (10 mg/liter), isoleucine (60 mg/liter), lysine (40 mg/liter), phenylalanine (60 mg/liter), threonine (50 mg/liter), methionine (10 mg/liter), and tryptophan (40 mg/liter)].

For lipidomics, cells were grown overnight to exponential phase [optical density at 600 (OD_600_) of 0.4 to 0.6], then were washed once in water, and snap-frozen in liquid nitrogen. For acyl-thiamine measurements, cultures were grown to the exponential phase overnight and then split in two; one half was left untreated, while the second half was supplemented with 100 µM thiamine. All cultures were then grown for 2 hours 30 min. Cells were washed once in water and snap-frozen in liquid nitrogen.

For growth assays on plates, yeast cells were grown to the exponential phase. Starting from a concentration of OD_600_ of 0.2, serial fivefold dilutions were spotted onto plates and incubated for 2 days.

### Yeast strain generation for protein purification

Yeast strain used for protein expression and purification was protease-deficient *S. cerevisiae* strain BJ2168 (MATα leu2 trp1 ura3-52 prc1-407 ptb1-1122 pep3-3). The protocol for generating BJ2168 lines expressing proteins of interest was followed as described in detail (*33*). Briefly, pYES2/CT C-HIS-YPR114w and pYES2/CT C-HIS-FLD-1 plasmids were transformed into competent BJ2168 hosts using lithium acetate/polyethylene glycol and heat-shock method. Transformed cells were streaked out onto selection agar plates (SC-Ura + 2% glucose) and incubated at 30°C for 48 to 72 hours. Individual colonies were picked and inoculated into 2 ml of SC-Ura + 2% glucose liquid media and incubated overnight at 30°C with shaking at 225 rpm. Glycerol stocks were prepared by mixing 750-μl overnight culture with 750 μl of 30% glycerol and were stored at −70°C.

### Immunoprecipitation of mitochondria

Mitochondrial immunoprecipitation (mito-IP) from HeLa control or TLCD1 KO 3XMyc-EGFP-OMP25 and 3XHA-EGFP-OMP25 stable cell lines was performed according to a published protocol with slight modifications (*31*). All mito-IP steps were conducted using ice-cold buffers either in a cold room or on ice, with all centrifugation steps performed at 4°C. Cells were washed twice with 30 ml of PBS, harvested in 1 ml of mito-IP buffer (10 mM KH_2_PO_4_ and 137 mM KCl), and collected at 700*g* for 5 min. The cells were then resuspended in 1 ml of mito-IP buffer containing protease inhibitors (78429, Thermo Fisher Scientific) per 15-cm plate and lysed with 25 passes through a Dounce homogenizer. The lysate was centrifuged at 1500*g* for 10 min to obtain a post-nuclear supernatant, which was incubated for 15 min with 100 μl of Pierce Anti-HA Magnetic Beads (88837, Thermo Fisher Scientific), washed, and pre-equilibrated in mito-IP buffer. Beads were collected using a magnetic rack, washed three times with 1 ml of mito-IP buffer for 5 min each, and dried beads were subsequently frozen in vials for lipid extraction. The 3XMyc-EGFP-OMP25 cell lines served as controls for nonspecific binding of HA beads, with any signal detected in these controls considered nonspecific.

### TLCD1 protein purification

Flp-In T-REx 293 cells with inducible expression of TLCD1 WT or mutant variants were seeded in 15-cm plates in a standard culture medium (for a typical purification, 10 plates were used). The next day, when cells were approximately 40% confluent, the expression was induced by adding tetracycline (1 μg/ml), and cells were incubated in the presence of tetracycline for 48 hours. Cells were then washed once with ice-cold PBS and scraped into ice-cold PBS on ice. The resulting cell suspension was centrifuged at 500*g*, 4°C for 5 min, before aspirating the PBS and storing cell pellet at −70°C.

To purify TLCD1, the cell pellet was defrosted, resuspended in 8.5 ml of Hepes buffer [20 mM Hepes (pH 7.4) and 150 mM NaCl], and solubilized by adding 1.5 ml of 10% LMNG (Anatrace) to achieve a final concentration of 1.5% LMNG. The mixture was incubated at 4°C for 2 hours. The suspension was then centrifuged at 128,000*g* for 1 hour at 4°C to separate the insoluble fraction. Meanwhile, 600 μl of Strep-Tactin XT 4 Flow beads slurry (Iba) was washed with 6 ml of Milli-Q water and 6 ml of wash buffer [20 mM Hepes, (pH 7.4), 150 mM NaCl, and 0.1% LMNG]. The clear supernatant was incubated with the washed beads for 2 hours to facilitate binding. After binding, the beads were washed twice with 6 ml each of wash buffer [20 mM Hepes (pH 7.4), 150 mM NaCl, and 0.1% LMNG] to remove unbound material. The protein was eluted using 1 ml of elution buffer [50 mM Hepes (pH 7.4), 150 mM NaCl, 0.05% LMNG, and 100 mM biotin]. Biotin was removed by passage through PD Minitrap G-25 columns (17085101, Cytiva). Protein concentration was then determined using NanoDrop ND-8000 spectrophotometer at 280 nm. The purified proteins were aliquoted and stored at −70°C until further analysis. All TLCD1 mutants were purified following the same protocol and were processed alongside the WT control.

To compare TLCD1 activity in different detergents, TLCD1 expression was induced in 293 cells as described above, and the cells from 40 15-cm plates were pooled and divided into four pellets. The same purification protocol was followed as outlined above, with LMNG replaced by the respective detergent at the same concentration. TLCD1 was purified in LMNG, DMNG, DDM, and GDN in parallel and reconstituted to the same concentration for in vitro assays.

### YPR114w and FLD-1 protein purification

To purify YPR114w and FLD-1 proteins, BJ2168 expression strains were taken from glycerol stocks and scaled up to 5 liters in YPG + 0.1% glucose medium at 30°C with shaking at 225 rpm. YPR114w expression was induced by adding galactose to a final concentration of 2%, and cells were collected 8 hours later by centrifugation. FLD-1 expression was induced by adding galactose to a final concentration of 0.4%, and cells were collected 4 hours later by centrifugation. Cell pellets were washed once with water and stored at −70°C.

To obtain membrane fractions, cell pellets were defrosted, resuspended in 100 ml of breaking buffer [0.65 M sorbitol, 100 mM tris-HCl (pH 8.0), 5 mM EDTA, 5 mM amino hexanoic acid, and 5 mM benzamidine hydrochloride] using a homogenizer and lysed using a Constant Systems Cell Disruptor (Constant Systems). Cell debris was removed by centrifugation (3000*g*, 20 min, 4°C). The pellet was discarded, and the supernatant was centrifuged at 125,000*g* for 1 hour at 4°C to obtain the membrane fraction. The membrane fraction was resuspended in wash buffer [0.65 M sorbitol, 100 mM tris-HCl (pH 7.4), 5 mM amino hexanoic acid, and 5 mM benzamidine hydrochloride] using a homogenizer and centrifuged again at 125,000*g* for 1 hour at 4°C. The pellet was resuspended in TBG buffer [100 mM tris-HCl (pH 7.4) and 10% glycerol] using a homogenizer and centrifuged at 125,000*g* for 1 hour at 4°C. The supernatant was discarded, and the pellet mass was measured. The pellet was finally resuspended in 10 ml of TBG buffer using a homogenizer, aliquoted, and stored at −70°C until further analysis.

To purify proteins, 5 ml of the membrane fractions was solubilized in a buffer containing 1.5% LMNG, 20 mM Hepes (pH 7.0), 150 mM NaCl, 30 mM imidazole, and 1X protease inhibitor cocktail to a final volume of 21 ml. This mixture was incubated for 1 hour at 4°C in Ti70 ultracentrifuge tubes. The insoluble material was pelleted by centrifugation at 118,000*g* for 45 min, and the supernatant containing the solubilized proteins was collected. Meanwhile, Ni-Sepharose excel resin (Cytiva) was washed with 6 ml of Milli-Q water and 6 ml of wash buffer A [20 mM Hepes (pH 7.0), 150 mM NaCl, 40 mM imidazole, and 0.2% LMNG]. The soluble fraction was then incubated with washed Ni-Sepharose excel resin for 2 hours to bind the His-tagged proteins. Post-binding, the resin was washed first with 15 ml of wash buffer A and subsequently with 3 ml of wash buffer B [20 mM Hepes (pH 7.0), 100 mM NaCl, and 0.2% LMNG]. The protein-bound resin was then resuspended in 1.5 ml of buffer B. Elution of the target proteins was achieved by incubating the resin overnight on a rotator at 4°C with 20 μg of Factor Xa protease and 2.5 mM CaCl_2_ to cleave and release the proteins. Protein concentration was then determined using NanoDrop ND-8000 spectrophotometer at 280 nm.

### CLN8 protein purification

HeLa cells were seeded in 15-cm plates in a standard culture medium (for a typical purification, 24 plates were used). The next day, when cells were approximately 40% confluent, cells were transfected with 300 μl of Opti-MEM, 6 μg of pcDNA3.1-C-HA CLN8 WT plasmid DNA, and 18 μl of FuGENE HD per plate. Cells were then cultured for 48 hours, washed once with ice-cold PBS, and scraped into ice-cold PBS on ice. The resulting cell suspension was centrifuged at 500*g*, 4°C for 5 min, before aspirating the PBS and storing cell pellet at −70°C.

To purify CLN8, the cell pellet was defrosted and resuspended in 5.1 ml of Hepes buffer [50 mM Hepes (pH 7.4) and 150 mM NaCl]. The cells were solubilized by adding 900 μl of 10% LMNG to achieve a final concentration of 1.5% LMNG and incubated at 4°C for 2 hours. The suspension was centrifuged at 128,000*g* for 1 hour at 4°C to separate the insoluble fraction. Meanwhile, 1 ml of Pierce Anti-HA Magnetic Beads (Thermo Fisher Scientific) slurry was washed three times (1 ml each) with wash buffer [20 mM Hepes (pH 7.4), 150 mM NaCl, and 0.1% LMNG] on a magnetic rack. The clear supernatant was then incubated with the washed anti-HA magnetic beads on a rotator for 4 hours at 4°C to facilitate binding. After binding, the beads were washed five times with 1 ml of wash buffer on a magnetic rack to remove unbound material. The protein was eluted overnight at 4°C using 1 ml of elution buffer [20 mM Hepes (pH 7.4), 150 mM NaCl, 0.05% LMNG, and HA peptide (2 mg/ml)]. The HA peptide was removed by passage through PD Minitrap G-25 columns. Protein concentration could not be accurately determined due to residual HA peptide presence in detergent micelles. The purified protein was aliquoted, snap-frozen in liquid nitrogen, and stored at −70°C until further analysis.

### Denaturing polyacrylamide gel electrophoresis (SDS-PAGE), in-gel fluorescence, Coomassie, and Western blotting

The 12% SDS–polyacrylamide gel electrophoresis (SDS-PAGE) gels were manually cast using Invitrogen SureCast Acrylamide Solution (40%). SDS-PAGE samples were prepared at room temperature without heating using Pierce Lane Marker Reducing Sample Buffer (to a final concentration of 2.5×). Notably, heating the samples resulted in all TLCD proteins forming high–molecular weight aggregates, similar to what is reported for other transmembrane proteins (*34*). Electrophoresis was performed in tris-glycine-SDS buffer at a constant voltage of 120 V for the stacking gel and 180 V for the resolving gel.

For Coomassie staining, gels were incubated with 15 ml of InstantBlue Coomassie Protein Stain (Abcam) overnight and then destained with water overnight. Stained gels were scanned using a stationary scanner.

To determine in-gel NBD fluorescence, 1.5 μg of the purified proteins was incubated with either 100 μM NBD-palmitoyl-CoA alone or with 100 μM NBD-palmitoyl-CoA and 500 μM 18:1-LPE at 30°C for 5 min. The reaction was stopped by adding Pierce Lane Marker Reducing Sample Buffer (to a final concentration of 2.5×). The proteins were then separated by SDS-PAGE as described previously. The in-gel fluorescent image was captured using the Amersham Typhoon imager (Cytiva) with the Cy2 fluorescence method. Subsequently, the gels were stained with Coomassie, and the total protein staining image was captured as described earlier.

For Western blotting, proteins were transferred onto a 0.45-μm PVDF membrane (Thermo Fisher Scientific) in chilled tris-glycine buffer (with 10% methanol) at a constant voltage of 90 V for 100 min using a Mini Trans-Blot system (Bio-Rad) with an ice pack. Membranes were blocked with 5% skimmed milk in 1X TBST, followed by overnight incubation with primary antibodies (anti-HA rabbit, 1:1000; anti-FLAG mouse, 1:1000; anti-tubulin rabbit, 1:1000 at 4°C; and anti-His horseradish peroxidase rabbit, 1:2000 at room temperature). This was followed by a 1-hour incubation with secondary antibodies (1:5000) at room temperature. Visualization was achieved by enhanced chemiluminescence (Thermo Fisher Scientific). Blots were exposed digitally using the ChemiDoc MP System (Bio-Rad). Where applicable, blots were presented as digital overlays of chemiluminescence and colorimetric images to show the molecular weight marker.

### Size exclusion chromatography

TLCD1 was initially purified as described previously, with biotin removed using a PD desalting column. Subsequently, the protein was concentrated using a 10-kDa MWCO spin column (UCF501008, Millipore) to achieve a final concentration of 0.5 mg/ml. A 100-μl aliquot of this concentrated protein solution was loaded onto an AKTA purifier system (GE Healthcare) equipped with a pre-equilibrated size exclusion column. The column was conditioned with SEC buffer [50 mM Hepes (pH 7.4), 150 mM NaCl, and 0.05% LMNG] before sample injection.

The AKTA system automatically collected fractions as the protein eluted from the column and measured absorbance at 280 nm. These fractions were then concentrated and analyzed using SDS-PAGE and in vitro activity assays.

### Cryo-EM grid preparation, data acquisition, and processing

For cryo-EM grid preparation, purified TLCD1 protein was concentrated to 1 mg/ml using Amicon Ultra 0.5 ml of 10-kDa centrifugal filter. A total of 3 μl of purified concentrated TLCD1 was frozen with Quantifoil R1.2/1.3 holey carbon grids using an FEI Vitrobot IV (Thermo Fisher Scientific) with blotting for 3 s at blotforce setting of −5. Screening images were taken using a Talos Arctica at 200 kV with a 100-μm objective aperture and 50-μm C2 aperture, and a Falcon 3 detector in linear mode. Data were collected in linear mode at 1.37 Å/pixel (nominal magnification of 73,000×) with a defocus range of −3.3 to −1.8 μm in 0.3-μm increments, with the autofocus routine running every 10 μm. Movie frames were collected at a nominal dose of 48.25 e−/Å^2^ with a 1.89-s exposure. Data were acquired as one shot per hole. Particles were picked using WARP (automated software for all pre-processing steps of cryo-EM data acquisition) (*35*) and then analyzed using CryoSPARC v3.3.2 (*36*).

### Acyltransferase activity assays

Enzymatic reactions for acyltransferase activity were set up in 1.5-ml microcentrifuge tubes with a total volume of 25 μl in Hepes buffer [20 mM Hepes (pH 7.4), 150 mM NaCl, and 0.05% LMNG]. Unless specified otherwise in the figure legend or below, the reaction mixture consisted of 3.4 nM enzyme, 100 μM substrate A (acyl-CoA), and 500 μM substrate B (thiamine or lysophospholipids). When an acyl-CoA mixture was used, the final concentration of each acyl-CoA was 100 μM. The reaction was prepared on ice and incubated in a water bath at 30°C for 15 min (unless otherwise indicated for time-course assays). The reaction was stopped by adding either 500 μl of butanol/methanol (3:1) for TLC detection or 500 μl of 80% acetonitrile (containing 0.2% formic acid) for LC-MS/MS detection.

To determine the *K*_m_ values for acyl-CoAs, the concentration of 18:1-LPE was kept constant at 250 μM. To determine *K*_m_ values for LPE or thiamine, the concentration of acyl-CoA was kept constant at 100 μM.

To assay TLCD1 in other detergents, TLCD1 preparations purified in different detergents were tested side by side under identical conditions as described above, with LMNG in the assay buffer replaced by the respective detergent at the same concentration (0.05%).

For CLN8 assays, reactions were performed in a 50-μl volume of Hepes buffer using 5 μl of purified protein, 100 μM acyl-CoA, and 500 μM 18:1-LPG. The reaction was prepared on ice and incubated in a water bath at 37°C for specified periods (up to 15 min). To prepare acyl-CoA for radiolabeling experiments, oleoyl [1-^14^C] CoA (American Radiochemicals) stock solution was mixed 1:5 with unlabeled oleoyl-CoA 10× 1 mM stock solution to produce a 10x radioactive tracer solution for assays.

In all assays, controls without enzyme, substrate A, or substrate B were included and presented in the figures where applicable. For time-course assays, the controls were incubated for the same duration as the longest time point. For varying concentration assays, the controls had the highest concentration.

### Lipid extraction

The extraction of total lipids from cellular matrices, immunoprecipitated mitochondria, and in vitro assays was conducted using the butanol-methanol (BUME) method, as described in detail (*37*). Note that the chloroform-methanol lipid extraction methods result in the partitioning of acyl-thiamines into the polar phase. A 2-ml screw cap plastic tubes were used (3469-11, Thermo Fisher Scientific), and a blank extraction was always performed in parallel to account for the plastic-related contaminants. The extraction commenced with the homogenization of frozen cell pellets, mitochondrial beads, or in vitro assay mixtures in a 0.5 ml of ice-cold solution of butanol to methanol in a 3:1 ratio. For lipidomics samples, BUME solution was enriched with SPLASH internal standard mix (330707, Avanti Polar Lipids). For the extraction, a further 0.5 ml of 1% acetic acid and 0.5 ml of a heptane:ethyl acetate 3:1 mixture were added, followed by vigorous vortexing for a total of 5 min. The mixture was then centrifuged at 6000*g* for 5 min, allowing for the separation of phases, after which the upper organic phase was carefully decanted into glass vials. A second extraction was conducted on the remaining aqueous phase, with the newly acquired upper phase being combined in the same glass vials as the first. Post-extraction, the solvents were evaporated under a stream of nitrogen, and the resultant dry lipid extracts were preserved at −70°C pending further analysis.

### Thin-layer chromatography (TLC)

Lipids were extracted from in vitro assays as described above and solubilized in 25 μl of high-performance liquid chromatography (HPLC)–grade chloroform. Subsequently, solubilized lipid extracts were carefully spotted at the base of 20 cm–by–20 cm TLC silica plates (Z292974, Sigma-Aldrich). Where applicable, phospholipid standards (approximately 1 μg) dissolved in chloroform were spotted alongside the experimental samples. These plates were then placed in hermetically sealed glass chambers containing a developing solvent composed of a 65:25:4 (v/v) mixture of chloroform, methanol, and water. The development of the plates proceeded until the solvent front reached approximately 2 cm below the top edge of the plate. Following development, the plates were dried under a laminar flow hood to remove any residual solvents. For fluorescence detection, the dried TLC plates were imaged using Amersham Typhoon imager with the Cy2 Fluorescence method. For phosphorimaging, TLC plates were incubated with pre-erased phosphor screens (GE Healthcare) in a light-proof cassette for 1 week. Phosphor screens were then imaged on Amersham Typhoon imager using Phosphor Imaging method. To visualize phospholipid standards, TLC plates were sprayed with 0.05% primuline (206865, Sigma-Aldrich) solution in 4:1 acetone:water. The plates were then dried overnight under laminar flow and imaged using an Amersham Typhoon imager with the Cy2 fluorescence method.

### Untargeted LC-MS lipidomics

Dried samples were reconstituted in 100 μl of isopropanol, acetonitrile, and water (2:1:1 ratio) and thoroughly vortexed. LC was conducted using a Shimadzu HPLC System, with 10 μl of the sample introduced onto a Waters Acquity Premier UPLC CSH column (1.7-μm pore size, 2.1 mm by 50 mm), which was maintained at 55°C. The mobile phase A comprised a 6:4 ratio of acetonitrile to water with 10 mM ammonium formate, and mobile phase B consisted of a 9:1 ratio of isopropanol to acetonitrile with 10 mM ammonium formate. A flow rate of 500 μl/min was maintained with a gradient protocol for mobile phase B as follows: 0.00 min_40% mobile phase B; 1.5 min_40% mobile phase B; 8.00 min_99% mobile phase B; 10.00 min_99% mobile phase B; 10.10 min_40% mobile phase B; 12.00 min_40% mobile phase. The sample injection needle was rinsed with a 9:1 isopropanol and acetonitrile solution (strong wash) and isopropanol, acetonitrile, and water (2:1:1, weak wash).

Mass spectrometric detection was carried out on a Thermo Fisher Scientific Q-Exactive Orbitrap equipped with a heated electrospray ionization source. The mass spectrometer was calibrated immediately before sample analysis using positive and negative ionization calibration solution (recommended by Thermo Fisher Scientific). The electrospray ionization parameters were optimized with a 50:50 mix of mobile phase A and B for spray stability, setting the capillary temperature at 300°C, and source heater temperature at 420°C, with the sheath, auxiliary, and spare gas flows at specific arbitrary units (40, 15, and 3, respectively), and source voltage at 4 kV. The mass spectrometer operated at a scan rate of 4 Hz, yielding a resolution of 35,000 at *m*/*z* 200, over a full-scan range from *m*/*z* 120 to 1800, with continuous switching between positive and negative modes.

For peak identification and mass spectra processing, Thermo Scientific Xcalibur Software was used. PC and PE species were quantified as [M+H]^+^ ions in positive mode, whereas cardiolipin (CL) and BMP species were quantified as [M−H]^−^ ions in negative mode. Acyl-thiamines were identified as [M]^+^ ions, as described in text and figure legends. Peak areas were normalized to class-specific internal standards, with the 15:0 to 18:1(d7) PG internal standard being used for CL and BMP quantification. To calculate the PC or PE composition as molar percentage, the molar abundance of each individual PC or PE species was divided by the sum of molar abundances of all measured PC or PE species.

### Targeted LC-MS/MS

Targeted analysis of acyl-thiamines, PEs, and BMPs were performed using a Xevo TQ-S triple quadrupole mass spectrometer (Waters, UK). Before analysis, samples were stored in glass vials at 8°C, and a volume of 10 μL from each sample was injected using an autosampler. Chromatographic separations were performed on an I-Class ACQUITY UPLC system equipped with a BEH C18 column (1 mm by 50 mm, 1.7-μm particle size, 130-Å pore size; Waters, UK) and maintained at 30°C. A 0.2-μm UPLC filter (Waters, UK) was used. The mobile phases were MS solvent A [5% ACN, 0.1% formic acid ( FA)] and B (90% ACN, 0.1% FA) at a flow rate of 0.2 ml/min with the following gradient: 0 to 1.5 min, 5% B; 1.5 to 4.5 min, 5 to 100% B; and 4.5 min until the end of the run, 100% B. The run times for the separations were 10 min for acyl-thiamine, 20 min for PE, and 60 min for BMP. The eluate was analyzed by MS using multiple reaction monitoring (MRM) with electrospray ionization in the positive ion mode. The source spray voltage was set to 3.1 kV, the desolvation temperature to 500°C, and the ion source temperature to 150°C. Cone voltages, collision energies, and specific MS/MS transitions for each analyte were programmed as detailed in the table S4. Nitrogen was used as the curtain gas and argon as the collision gas. Quantification was performed using MassLynx 4.1 software, with automatic peak selection followed by manual verification and adjustment where necessary. To quantify PE, we established a standard curve by creating a five-point series with 10-fold serial dilutions of the 18:1-18:1 PE standard (Avanti). This curve was analyzed concurrently with each batch of samples to ensure accurate quantification. Acyl-thiamine and BMP abundances are presented as arbitrary units.

### Confocal microscopy

Cells were fixed in 5% paraformaldehyde in PBS at 37°C for 15 min, then washed three times with PBS, and quenched with 50 mM ammonium chloride in PBS. After three more washes in PBS, cells were permeabilized in 0.1% Triton X-100 in PBS for 10 min, followed by another three washes in PBS. Cells were blocked with 10% FBS in PBS, incubated with primary conjugated antibodies (1:500 dilution) in 5% FBS in PBS overnight at 4°C, and then washed three times in 5% FBS in PBS. Coverslips were briefly rinsed in water and mounted onto slides using ProLong Diamond Antifade Mountant (P36935, Thermo Fisher Scientific). Slides were imaged using a 100× objective lens (numerical aperture 1.4) on a Nikon Eclipse TiE inverted microscope with appropriate lasers using an Andor Dragonfly 500 spinning disk system equipped with a Zyla 4.2 PLUS sCMOS camera (Andor) and coupled with Fusion software.

### Molecular docking simulations

Blind docking simulations were conducted using AutoDock Vina (version 1.2.5) (*38*). The docking grids were defined as cubic boxes with dimensions of 80 Å on each side, fully encompassing the entire protein structures, which were treated as rigid bodies. Protein structures for human TLCD1 (UniProt ID: Q96CP7) and human CLN8 (UniProt ID: Q9UBY8) were sourced from AlphaFold2 (*39*–*41*). The C-terminal nonstructural regions of CLN8 were truncated to minimize the formation of artifactual binding sites.

The ligands thiamine and 18:1 lyso-PE (1-oleoyl-2-hydroxy-sn-glycero-3-phosphoethanolamine) and 18:1 lyso-PG [1-oleoyl-2-hydroxy-sn-glycero-3-phospho-(1′-rac-glycerol)] were docked into TLCD1 and CLN8, respectively. Specifically, thiamine and 18:1 Lyso-PE were docked into TLCD1, while 18:1 Lyso-PG targeted CLN8. Hits were selected on the basis of the lowest binding free energies exhibited, which were −6.7 kcal/mol for thiamine, −13.7 kcal/mol for 18:1 Lyso-PE, and −11.6 kcal/mol for 18:1 Lyso-PG.

### Protein sequence alignment and phylogenetic analysis

The following UniProt protein sequences were used for alignment: TLCD1 (Q96CP7), TLCD2 (A6NGC4), TLCD3A (Q8TBR7), TLCD3B (Q71RH2), TLCD4 (Q96MV1), TLCD5 (Q6ZRR5), CLN8 (Q9UBY8), CERS6 (Q6ZMG9), YPR114w (Q06107), YJR116w (P47153), FLD-1 (G5EF48), CERS1 (P275440, CERS2 (Q96G23), CERS3 (Q8IU89), CERS4 (Q9HA82), CERS5 (Q8N5B7), TRAM1 (Q15629), TRAM1L1 (Q8N609), and TRAM2 (Q15035). Protein sequences were aligned using SnapGene v7.2 software using the Clustal Omega method with default settings. Subsequently, phylogenetic trees were constructed using TreeViewer v2.2 software (*42*). The trees were generated using the Neighbor-Joining method, with evolutionary distances calculated on the basis of the BLOSUM62 matrix.

### Statistical analysis, data visualization, and reproducibility

All experimental data are presented as means, with error bars indicating the SEM. The number of replicates involved is specified in the figure legend. When applicable, data are also expressed as fold changes relative to a specified control value, which is detailed in the legend. For in vitro assays, each data point represents a single measurement. The statistical methods used are outlined in the legend. For comparing two groups, a Student’s *t* test was used. For comparisons involving more than two groups, a one-way analysis of variance (ANOVA) was applied. In cases where more than one factor influenced the variable under study, a two-way ANOVA was performed to assess the impact of each factor and their interaction. Either Sidak’s or Dunnett’s post hoc tests were used for multiple comparisons, as specified in figure legend.

All in vitro experiments involving purified proteins—including cryo-EM, biochemical, and enzymatic assays—were replicated at least once using a different batch of purified protein to ensure reproducibility. Each batch of protein was produced from the same stable cell line but was cultured on separate occasions to constitute separate biological replicates. For CLN8 purification, a separate transfection was performed to validate the reproducibility of the findings. For all in vitro assays, the results from each biological replicate were consistent. Consequently, only one representative graph is presented for each set of experiments.

All statistical analyses, graph generation, and enzyme kinetics evaluations were conducted using GraphPad Prism version 10.2. Figures and graphs were further refined and edited for presentation using Affinity Publisher version 2.4.2. SnapGene version 7.2 was used to produce visual protein sequence alignments and gene editing maps. In addition, graphic schematics incorporated in the figures were sourced from Bioicons.com.

## Supplementary Materials

### The PDF file includes:

Figs. S1 to S17 Tables S1 to S4 Legends for data S1 to S4 References
