# Supplementary material for: TRAM-LAG1-CLN8 family proteins are acyltransferases regulating phospholipid composition
Source: Sci Adv. 2025 Feb 19;11(8):eadr3723. doi: 10.1126/sciadv.adr3723 (PMC11838012; doi:10.1126/sciadv.adr3723)
Supplement: Supplementary file 1 — Figs. S1 to S17 Tables S1 to S4 Legends for data S1 to S4 References [file sciadv.adr3723_sm.pdf]

Science Advances
AAAS

Supplementary Materials for

TRAM-LAG1-CLN8 family proteins are acyltransferases regulating phospholipid composition

Pradeep K. Sheokand *et al.*

Corresponding author: Kasparas Petkevicius, kp416@mrc-mbu.cam.ac.uk

Sci. Adv. **11**, eadr3723 (2025)
DOI: 10.1126/sciadv.adr3723

The PDF file includes:

Figs. S1 to S17
Tables S1 to S4
Legends for data S1 to S4
References

Other Supplementary Material for this manuscript includes the following:

Data S1 to S4

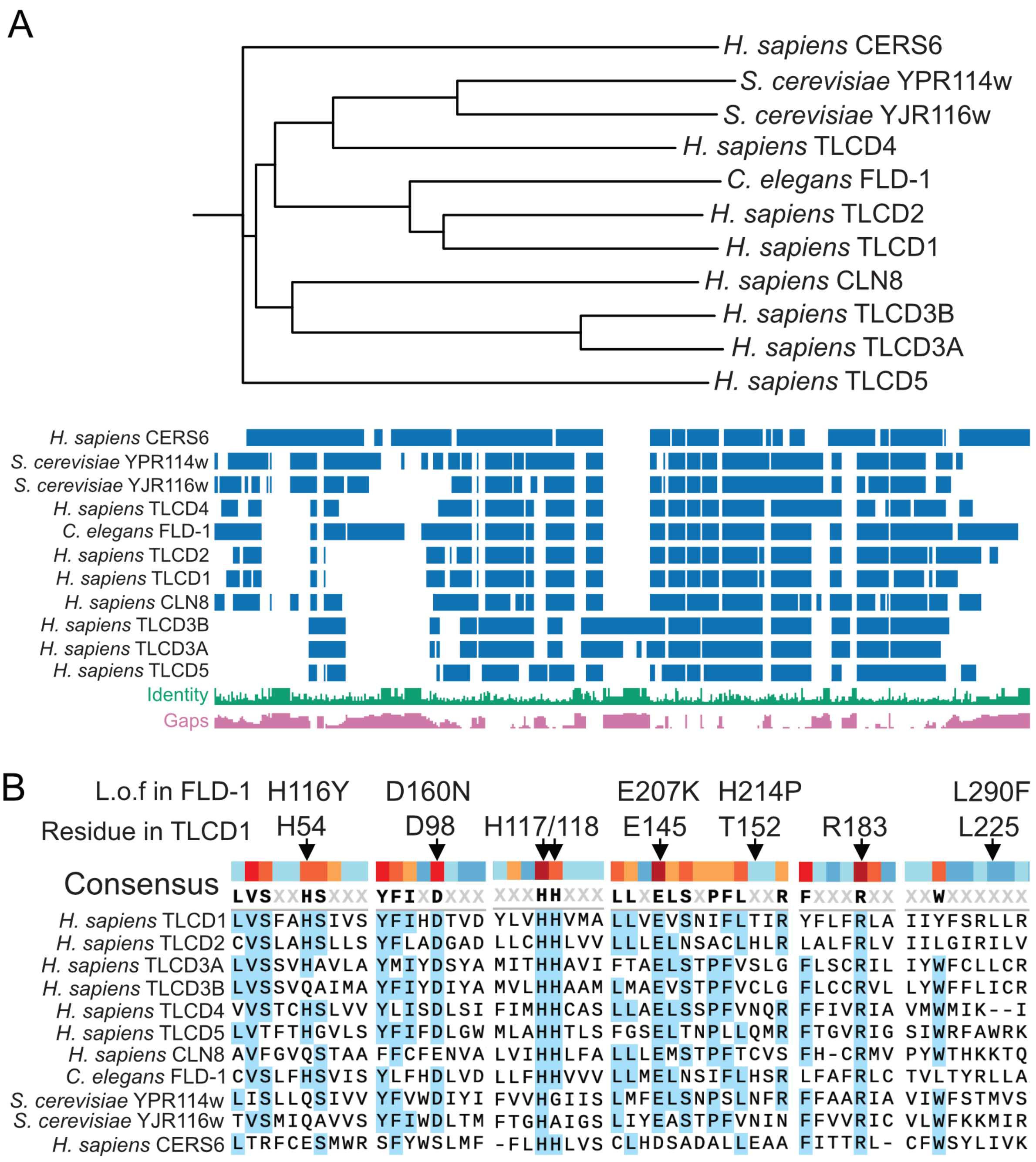

Fig. S1. Evolutionary relationship between TLCD family members across species.

A. Neighbor-joining phylogenetic tree of TLCD family proteins related to the present study. Protein sequence alignment used to generate the tree is illustrated below. **B.** Protein sequence alignment of indicated TLCD family proteins. Only the regions surrounding conserved residues are shown. Arrows above indicate conserved residues related to the present study, and denote the residues identified to cause loss of function (L.o.f.) in FLD-1 in a previous *C. elegans* genome-wide screen (*1*).

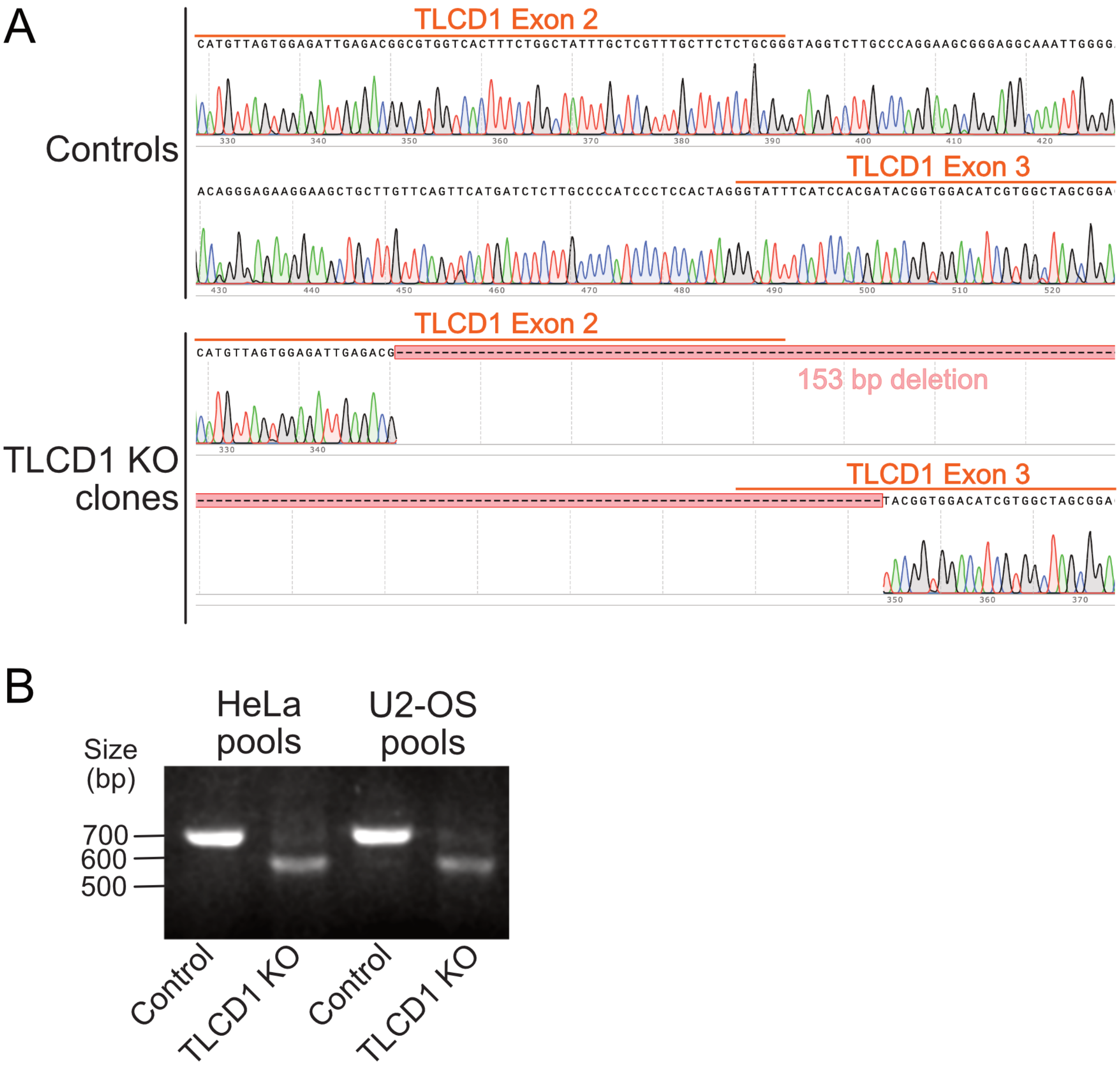

Fig. S2. Generation and validation of TLCD1 KO cell models.

A. Representative Sanger sequencing traces obtained from control and TLCD1 KO HeLa and U-2OS clones. **B.** PCR analysis of CRISPR-targeted locus of HeLa and U-2OS control and TLCD1 KO pools. The 1 kb DNA ladder band positions are indicated on the left. Note that in our dual gRNA targeting strategy, each gRNA may independently induce a deletion of up to a few base pairs. However, the resulting TLCD1 frameshift would yield a PCR band indistinguishable from the control, regardless of the presence a small deletion.

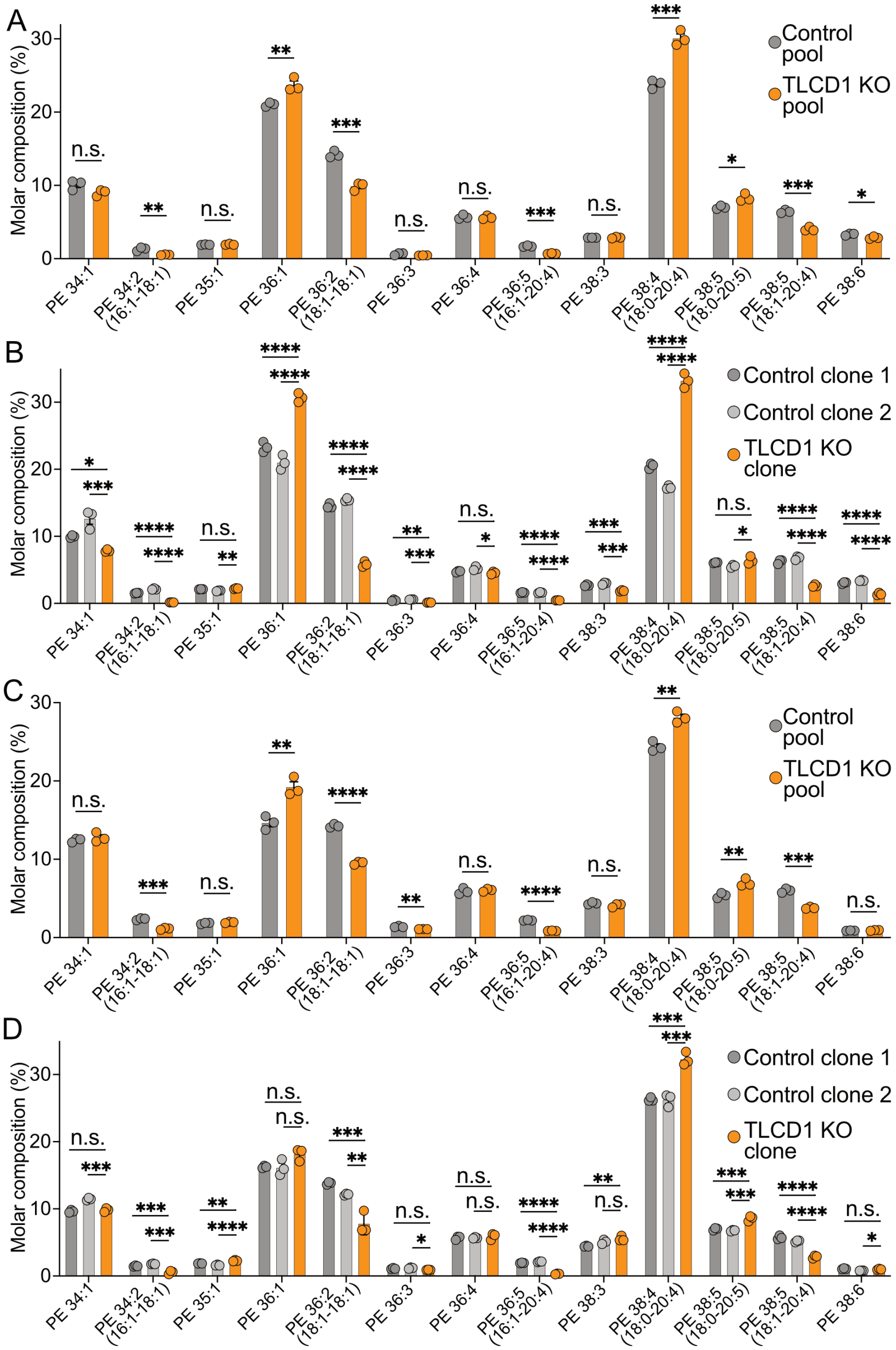

Fig. S3. PE molar composition of TLCD1 KO HeLa and U-2OS cell models.

The molar composition of PE species, calculated by dividing the abundance of each indicated PE species by total measured PE amount, in control or TLCD1 KO (**A**) HeLa pools; (**B**) HeLa clones; (**C**) U-2OS pools; (**D**) U-2OS clones. N=3 replicates in each analysis. N.s– not significant, *- $p<0.05$, **- $p< 0.01$, ***- $p< 0.001$ and ****- $p< 0.0001$ using (**A** and **C**) two-tailed unpaired student's t-test, or (**B** and **D**) one-way ANOVA with Sidak's post-hoc test. Numbers in brackets indicate the most likely inferred identities of individual acyl chains. All data were obtained in an untargeted manner.

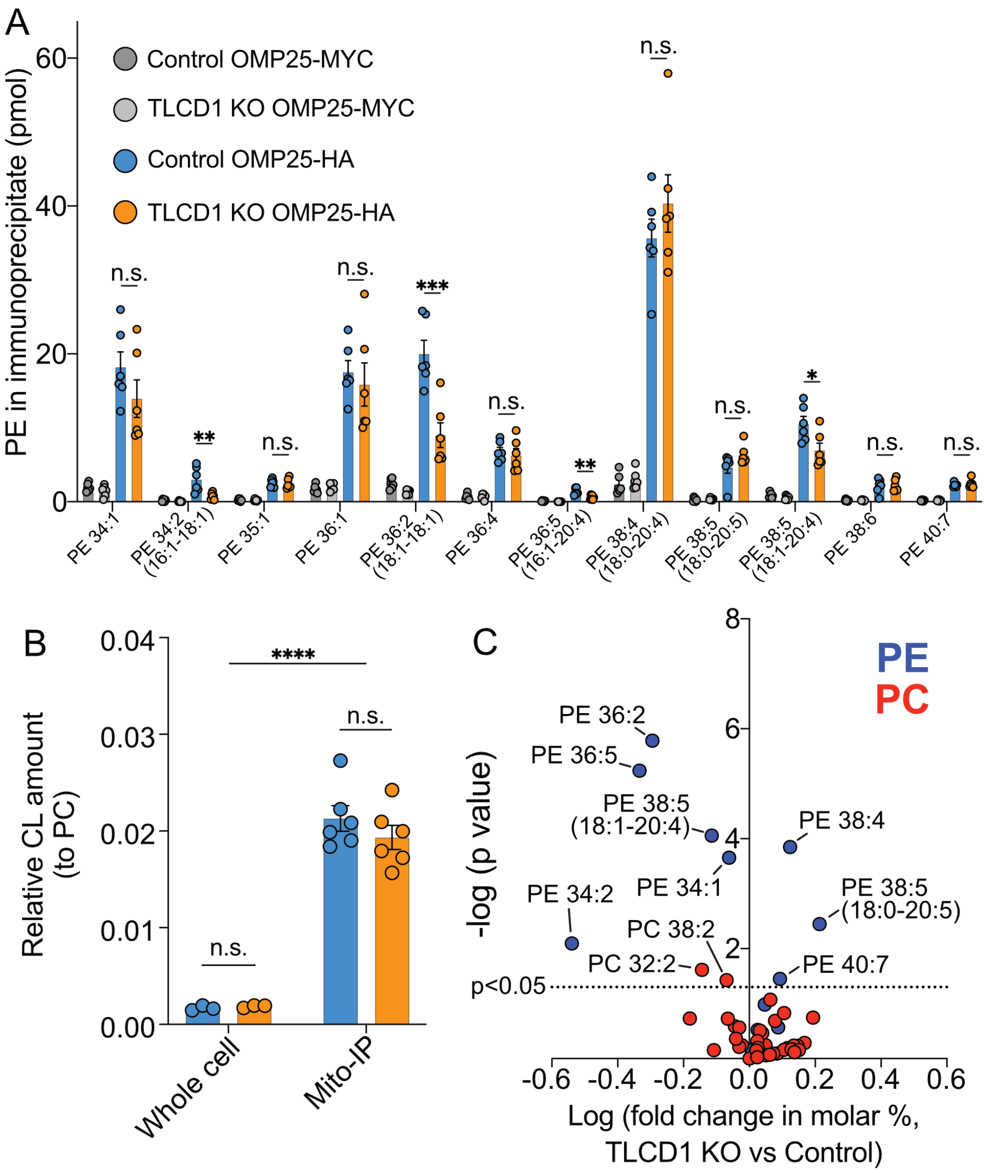

Fig. S4. Mitochondrial PE composition in HeLa TLCD1 KO cells.

A. Levels of indicated PE species measured in anti-HA immunoprecipitates of HeLa control or TLCD1 KO pools, stably expressing either MYC- or HA-OMP25 protein (n=6 paired replicates per condition). Cells expressing MYC-OMP25 are used as controls to account for non-specific binding to the anti-HA beads, with signal detected in MYC-OMP25 conditions attributed to non-specific immunoprecipitation. **B.** Cardiolipin (CL) enrichment, calculated by dividing total measured CL levels to total measured PC levels in each sample, in HA-OMP25-expressing HeLa control or TLCD1 KO whole cells (n=3) or anti-HA immunoprecipitates (n=6). **C.** Volcano plot comparing PE and PC species measured in anti-HA immunoprecipitates of HA-OMP25-expressing HeLa control or TLCD1 KO whole cells (n=6). N.s– not significant, *- p<0.05, **- p< 0.01, ***- p< 0.001 and ****- p< 0.0001 using (**A** and **C**) two-tailed paired student's t-test, or (**B**) two-way ANOVA with Sidak's post-hoc test. Numbers in brackets indicate the most likely inferred identities of individual acyl chains. All data were obtained in an untargeted manner.

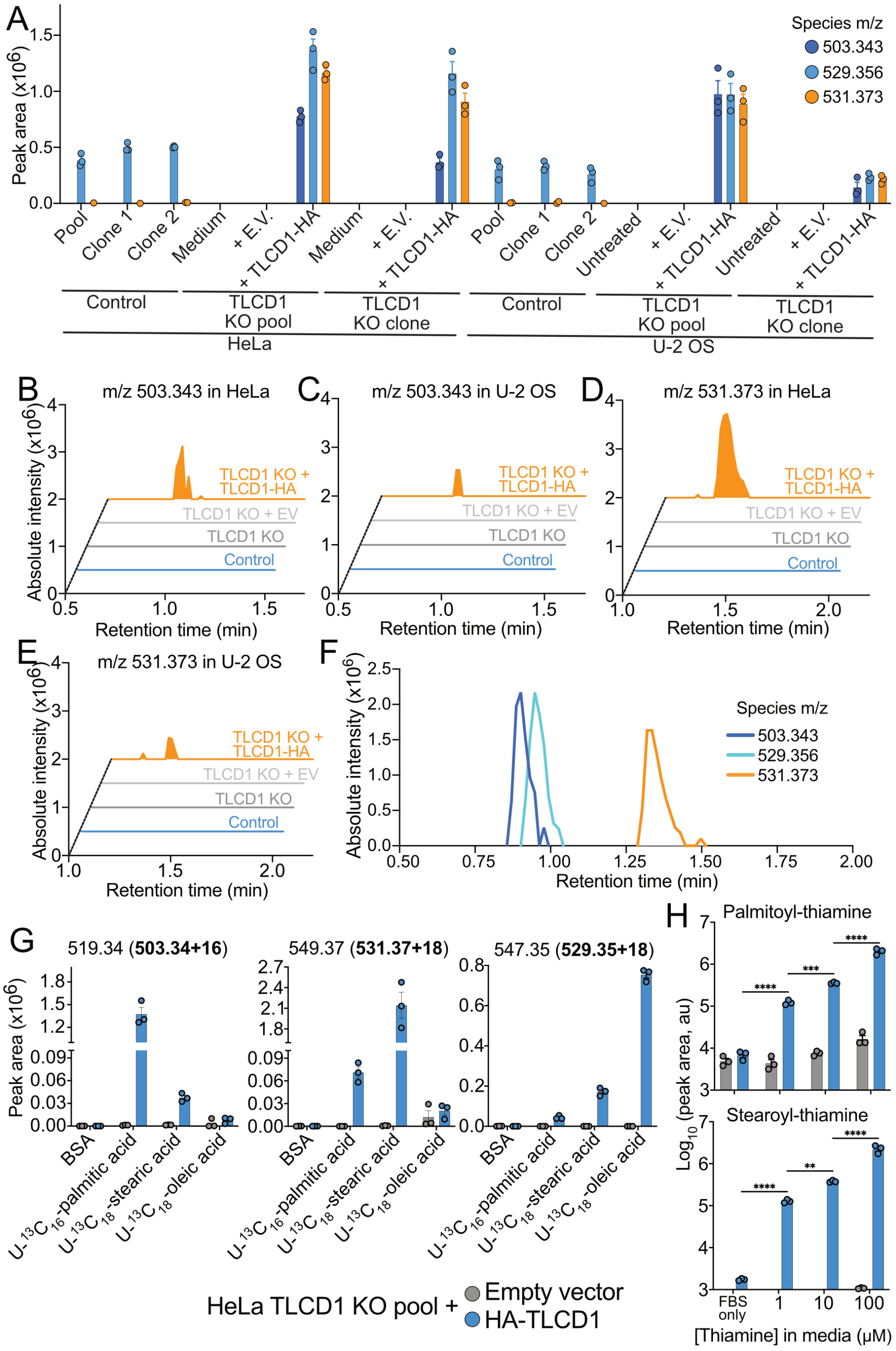

Fig. S5. Acyl-thiamine species measured in cell culture models.

A. The abundance of m/z 503.34, 529.35 and 531.37 ions in different cell models and conditions, as indicated below the graph (n=3). Representative aligned chromatograms of (**B** and **C**) m/z 503.34 ions and (**D** and **E**) m/z 531.37 ions measured in HeLa and U-2OS control and TLCD1

KO clones, transfected with either empty vector (EV) or TLCD1-HA plasmids. **F.** Representative chromatogram trace comparing the retention times of m/z 503.34, 529.35 and 531.37 ions in HeLa TLCD1 KO clone transfected with TLCD1-HA plasmid. **G.** The abundance of indicated m+16 and m+18 species (indicative of respective U-^{13}C fatty acid labeling), in TLCD1 KO HeLa pool, transfected with EV or TLCD1-HA, and treated with either BSA alone or BSA-conjugated U-^{13}C fatty acids (n=3). Note that in cells, stearic acid can be desaturated to oleic acid, retaining the U-^{13}C carbon labeling. **H.** The abundance of palmitoyl- and stearoyl-thiamine in HeLa TLCD1 KO pool transfected with TLCD1-HA, cultured in varying amounts of thiamine as indicated (n=3). In **H**, **- $p< 0.01$, ***- $p< 0.001$ and ****- $p< 0.0001$ using one-way ANOVA with Sidak's post-hoc test. Data were obtained in (**A** to **F**) an untargeted, and in (**G** and **H**) a targeted manner.

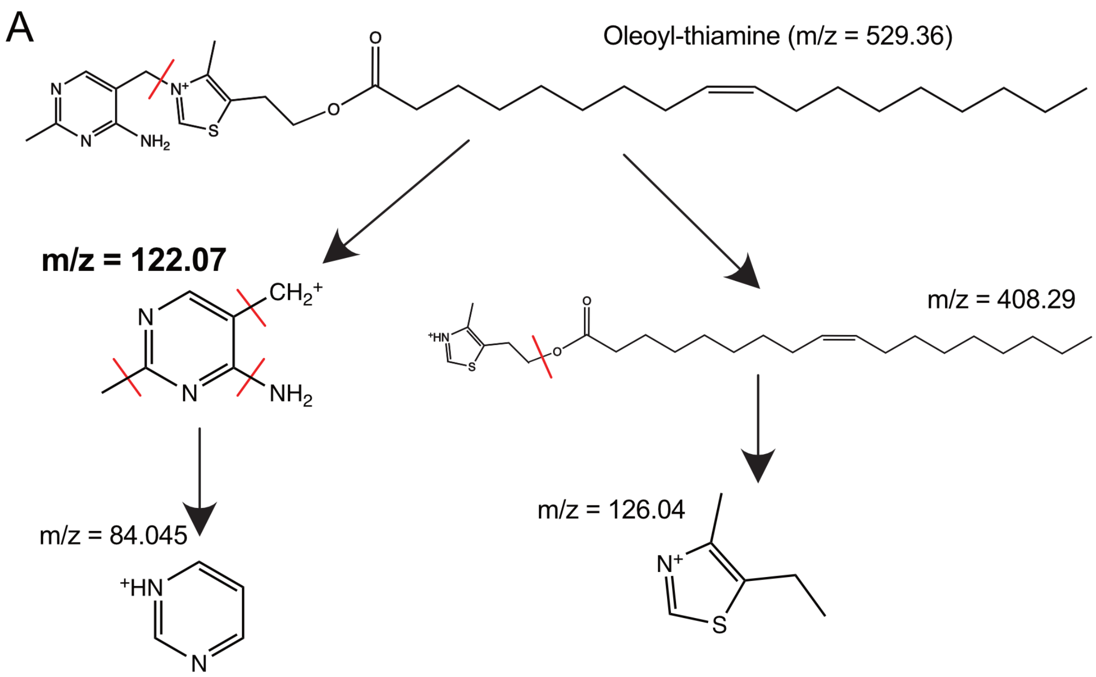

B

	Thiamine-d3	U-$^{13}C_{18}$-oleic acid
**Labelling**	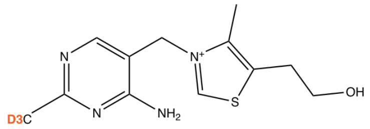	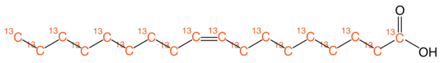
**Precursor m/z**	532.36 (529.36 + 3)	547.42 (529.36 + 18)
**Observed fragment m/z**	408.29 (unlabelled)<br>126.04 (unlabelled)<br>125.07 (122.07 + 3)<br>84.045 (unlabelled)	426.35 (408.29 + 18)<br>126.04 (unlabelled)<br>122.07 (unlabelled)<br>84.045 (unlabelled)
**Not detected fragment m/z**	411.29 (408.29 + 3)<br>129.04 (126.04 + 3)<br>122.07 (unlabelled)<br>87.045 (84.045 + 3)	408.29 (unlabelled)

Fig. S6. Elucidation of acyl-thiamine structure by stable labeling and fragmentation.
A. Proposed fragmentation tree of oleoyl-thiamine in a positive detection mode. Thiamine and oleate ester, guided by previous models of thiamine fragmentation (*43*), is the only conjugate between thiamine and oleic acid that is compatible with the observed fragment ions. Red lines indicate the bond break points. Fragment indicated in bold (122.07) was used for targeted quantification of acyl-thiamine species in this study. **B.** Validation of proposed oleoyl-thiamine structure by stable labeling and targeted MS/MS analysis of indicated fragment ions. Fragments that were observed and those that were monitored but could not be detected are listed.

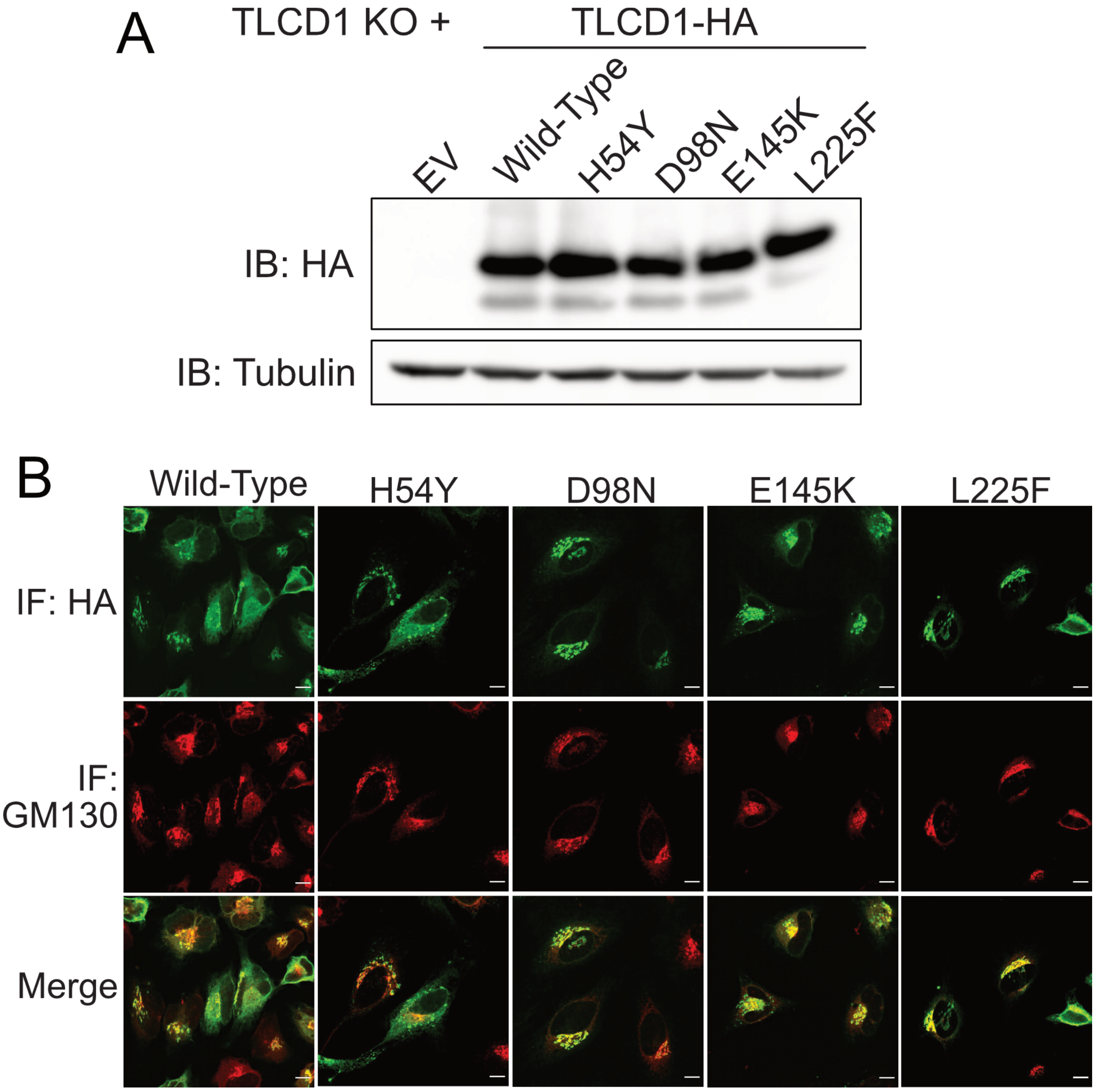

Fig. S7. Expression and localization of TLCD1 mutant proteins.

A. Western blot of HA-tag and Tubulin loading control in TLCD1 KO HeLa cells, transfected with EV or plasmids encoding TLCD1-HA variants. **B.** Representative immunofluorescence images of TLCD1 KO HeLa cells, transfected with EV or plasmids encoding TLCD1-HA variants, and stained for HA tag (green) and GM130 (Golgi marker, red). Yellow – merged channels. White bar = 10 μm.

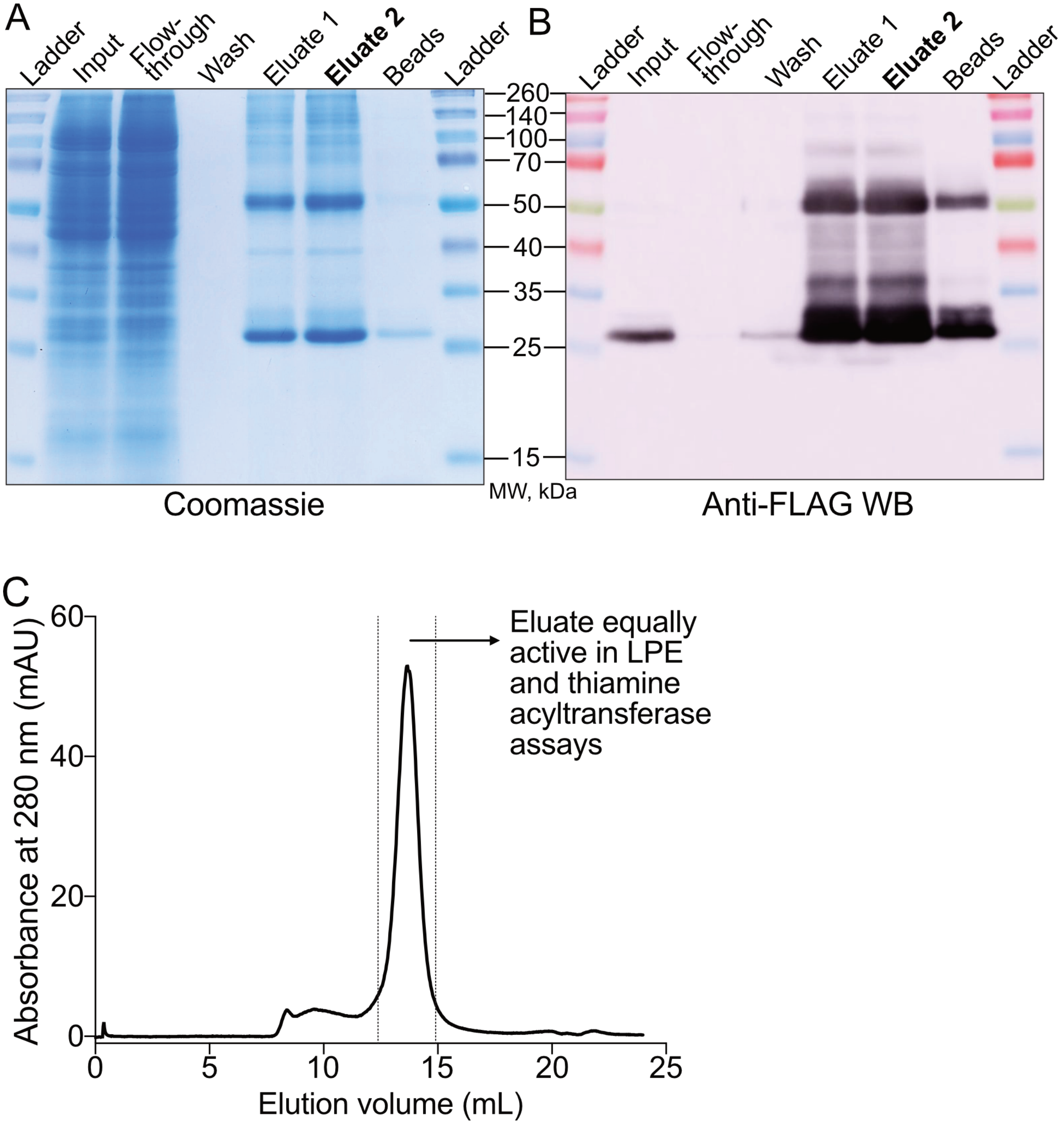

Fig. S8. Purification of TLCD1 from 293 cell line.

A. Total protein Coomassie staining and (**B**) Western blot of FLAG tag in the indicated fractions obtained during FLAG-STREP-tagged TLCD1 purification using Strep-Tactin sepharose. Eluate 1 represents the initial elution from beads using biotin, and eluate 2- size-exclusion column cleanup of biotin from purified TLCD1. Eluate 2 was used in the *in vitro* assays. Note that high molecular weight bands are SDS-resistant TLCD1 aggregates resulting from denaturation. The Western blot image is presented as a chemiluminescence and colorimetric overlay with molecular weight markers indicated. **C.** Size-exclusion chromatography (SEC) of purified TLCD1, showing the presence of a single protein population. The enzymatic activity of the SEC-collected eluate matches that of Eluate 2 from **A** (data not shown).

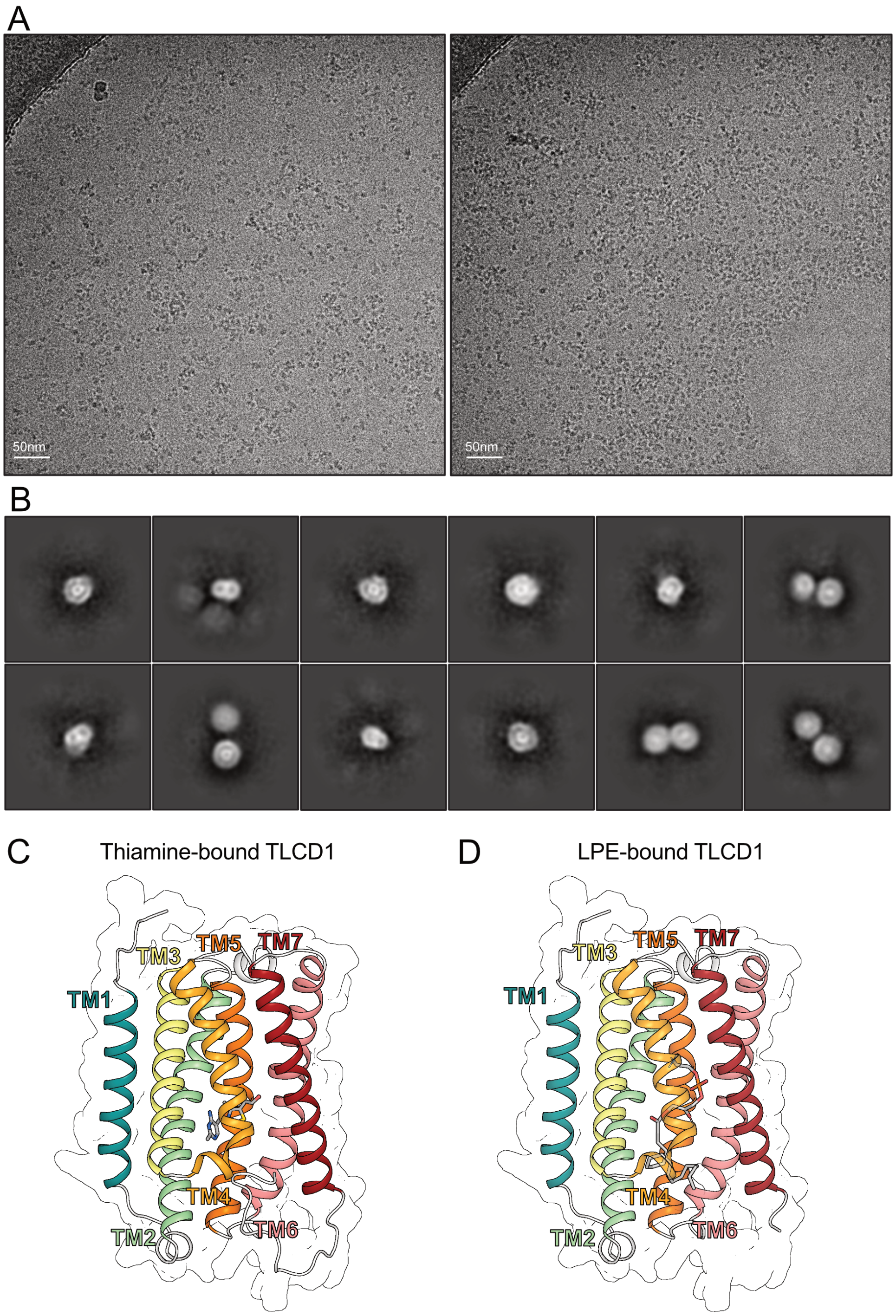

Fig. S9. Structural characterization and molecular docking simulations of TLCD1 protein. **A.** Collected micrographs of the TLCD1 sample. **B.** Representative 2D classifications showing top and side views of the protein. Molecular docking of (**C**) thiamine and (**D**) 18:1-LPE to the AlphaFold2-predicted structure of TLCD1, showing the whole protein from the side. Note that AlphaFold2 model confidence is very high (pLDDT > 90) and the predicted structure closely resembles the reported structure of the TLCD1 paralogue CERS6 (*11*).

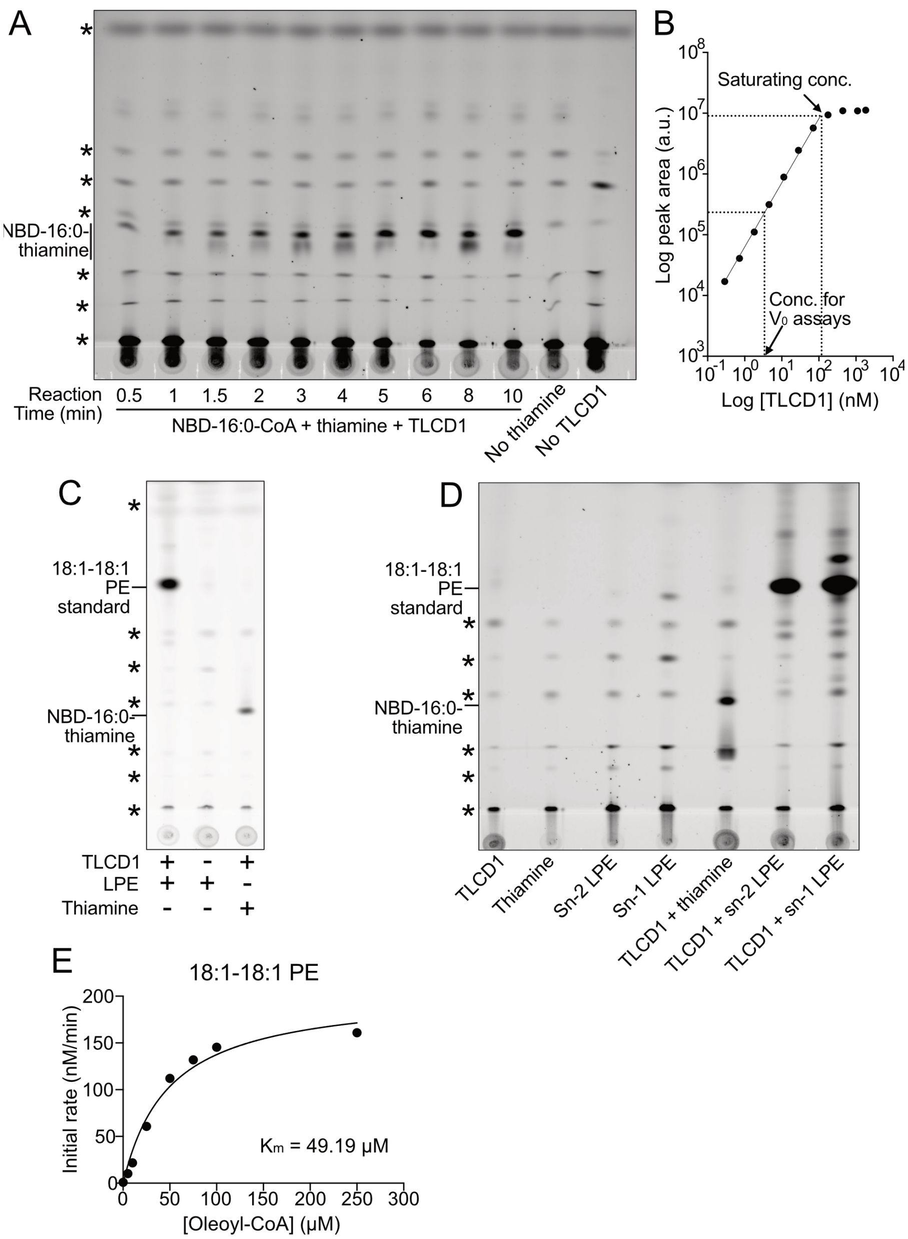

Fig. S10. Enzymatic characterization of TLCD1 protein.

A. Fluorescence imaging of thin-layer chromatography (TLC) separation of time-course assays with NBD-palmitoyl-CoA, thiamine, and TLCD1. **B.** Logarithmic plot of oleoyl-thiamine abundance in assays with oleoyl-CoA, thiamine, and increasing TLCD1 concentrations. **C.** Fluorescence TLC imaging of assays with NBD-palmitoyl-CoA, thiamine, LPE, and TLCD1; or (**D**) of assays with different LPE isoforms. **E.** Michaelis-Menten plot of initial reaction rates (V_0) in assays with purified TLCD1, 18:1-LPE, and increasing oleoyl-CoA concentrations. Non-specific bands in NBD-palmitoyl-CoA TLC are marked with '*'. Migration of NBD-palmitoyl-thiamine products and 18:1-18:1 PE standards are indicated. All data shown are representative of at least two independent protein purifications. All MS/MS data were obtained in a targeted manner.

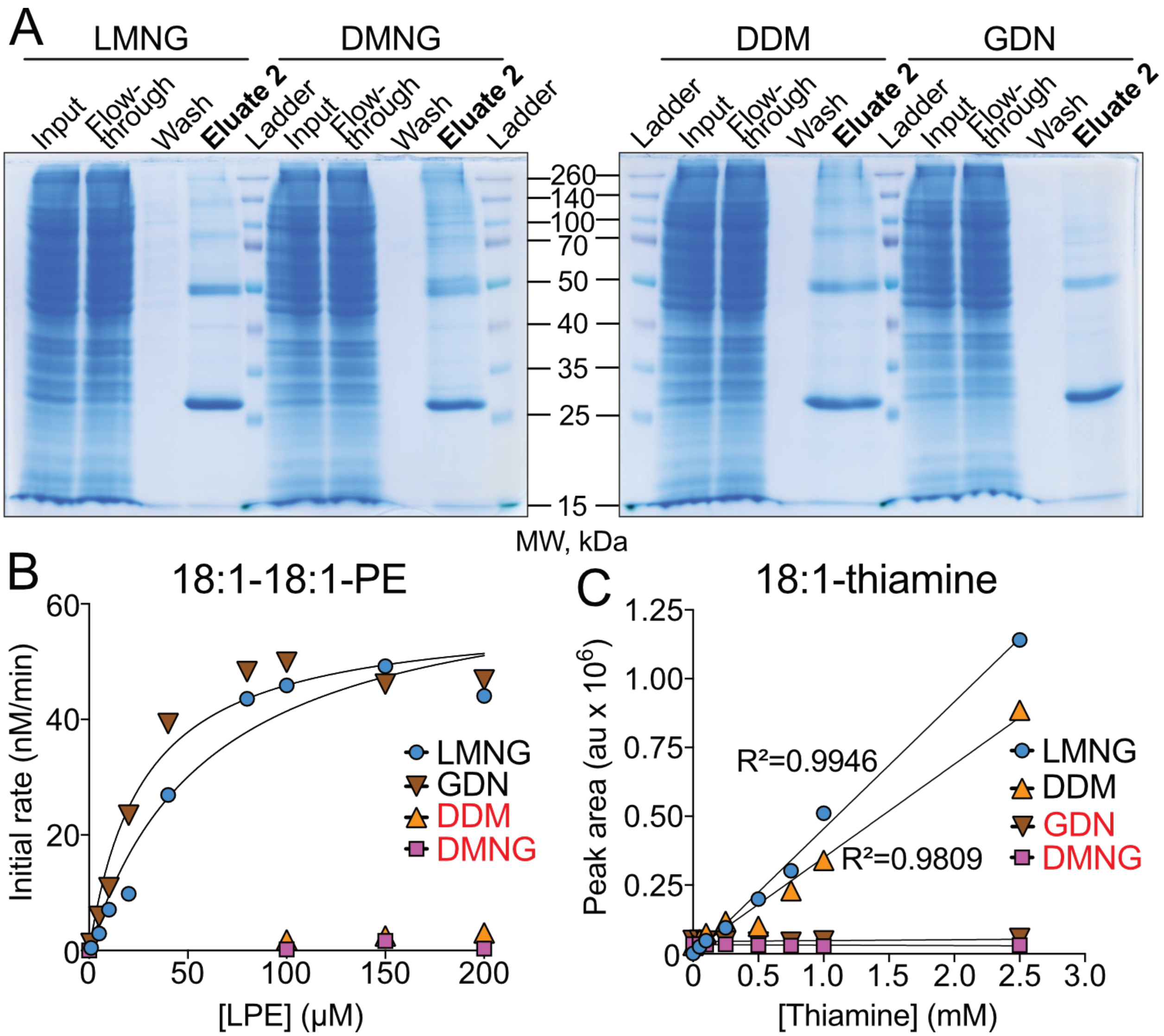

Fig. S11. Comparison of TLCD1 enzymatic activity in different detergents.

A. Coomassie staining of total protein in the specified fractions collected during the purification of FLAG-STREP-tagged TLCD1 using Strep-Tactin Sepharose, conducted in the presence of the indicated detergents. Eluate 2 represents size-exclusion column cleanup of biotin from purified TLCD1. Eluate 2 was used in the *in vitro* assays. Note that high molecular weight bands are SDS-resistant TLCD1 aggregates resulting from denaturation. **B.** Michaelis-Menten plot of V_0 assays conducted in different detergents with oleoyl-CoA, increasing 18:1-LPE concentrations and TLCD1 purified in those detergents as indicated. For LMNG, K_m = 60.65 µM and k_{cat} = 19.57 min^{-1}. For GDN, K_m = 27.29 µM and k_{cat} = 17.19 min^{-1}. TLCD1 was inactive in DMNG and DDM detergents. **C.** Linear regression between thiamine concentration and the oleoyl-thiamine synthesis rates in V_0 assays using TLCD1 purified in indicated detergents with oleoyl-CoA and thiamine, with assays conducted in the corresponding detergents. TLCD1 was inactive in DMNG and GDN detergents.

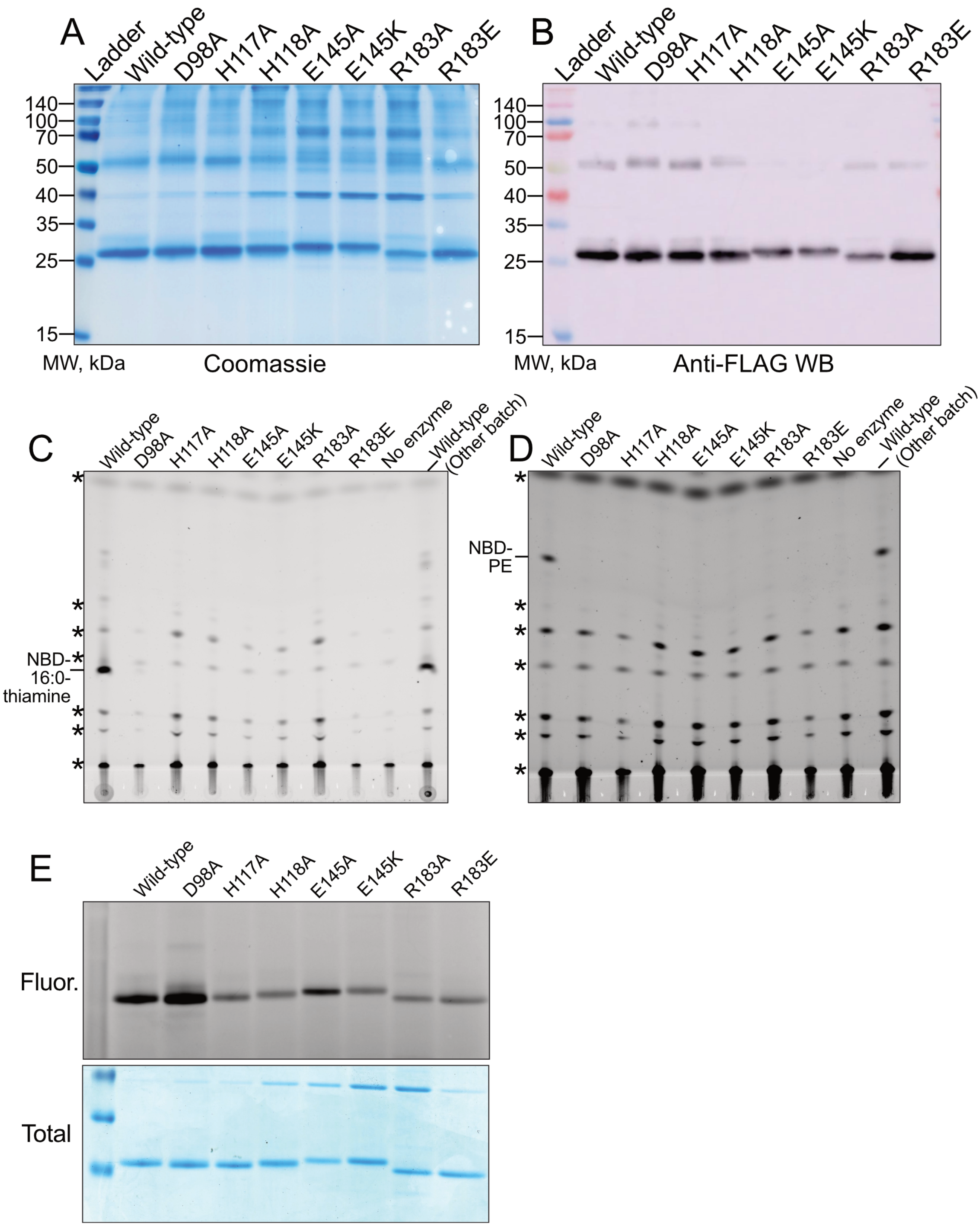

Fig. S12. Purification and enzymatic characterization of TLCD1 mutant proteins.

A. Total protein Coomassie staining and **(B)** Western blot of FLAG tag of the final eluates from FLAG-STREP-tagged TLCD1 wild-type and indicated mutant purifications using Strep-Tactin sepharose. The Western blot image is displayed as an overlay of chemiluminescence and colorimetric images, with molecular weight markers indicated. **C.** Fluorescence imaging of thin-layer chromatography (TLC) separation for assays with NBD-palmitoyl-CoA, indicated TLCD1

variants and thiamine or (**D**) LPE. **E.** In-gel NBD fluorescence (top panel) and total protein Coomassie staining (bottom panel) of purified TLCD1 variants, incubated with NBD-16:0-CoA for 5 min as indicated. Non-specific bands in NBD-palmitoyl-CoA TLC are marked with '*'. Migration of NBD-palmitoyl-thiamine products and 18:1-18:1 PE standards are indicated. All data shown are representative of two independent protein purifications.

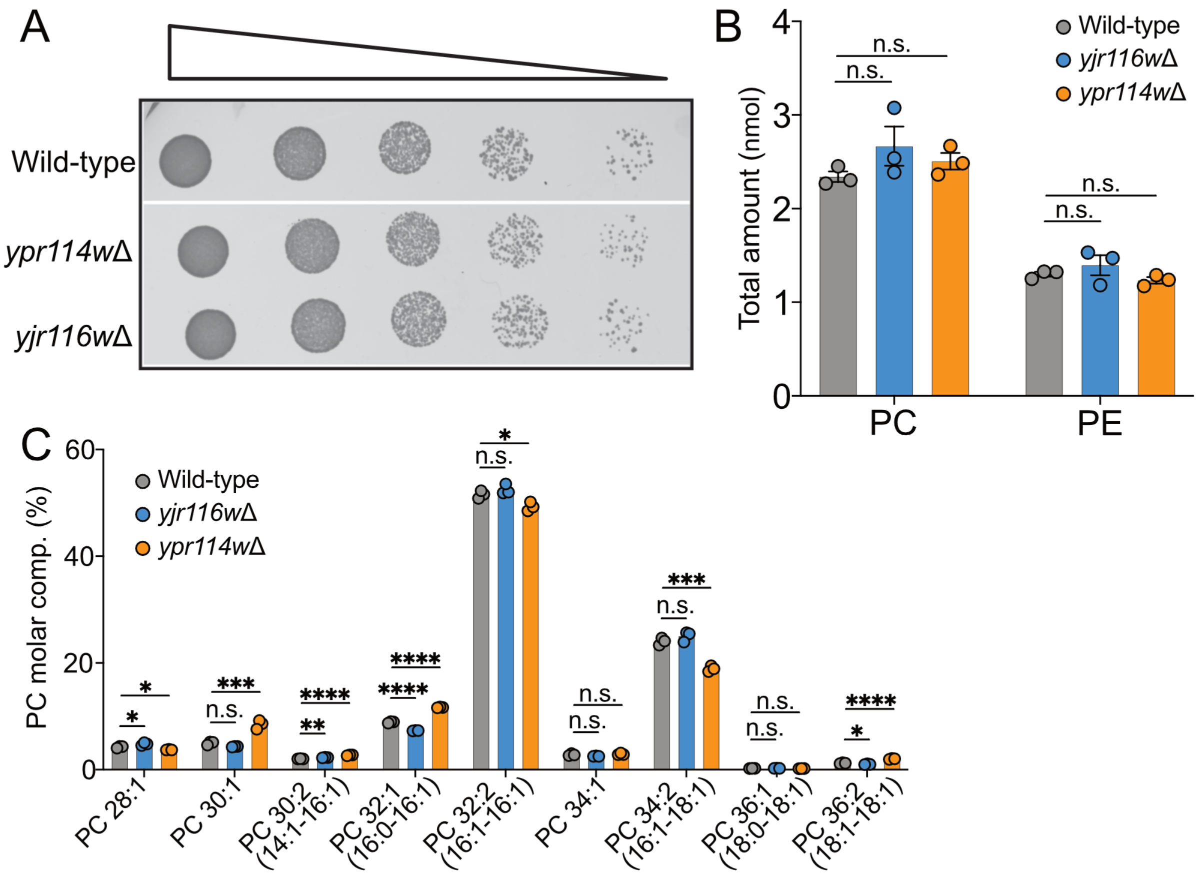

Fig. S13. Characterization of *S. cerevisiae ypr114w* and *yjr116w* mutant strains.

A. The growth of wild-type, *ypr114w*Δ or *yjr116w*Δ *S. cerevisiae* strains, spotted in serial 5-fold dilutions. **B.** The sum of total measured PC and PE species and (**C**) PC molar composition of the wild-type, *ypr114w*Δ or *yjr116w*Δ *S. cerevisiae* strains (n=3). N.s.- not significant, *- p< 0.01, **- p< 0.01, ***- p< 0.001 and ****- p< 0.0001 using (**B**) two-way ANOVA and (**C**) one-way ANOVA with Dunnett's post-hoc tests. Data in (**B** and **C**) were obtained in an untargeted manner.

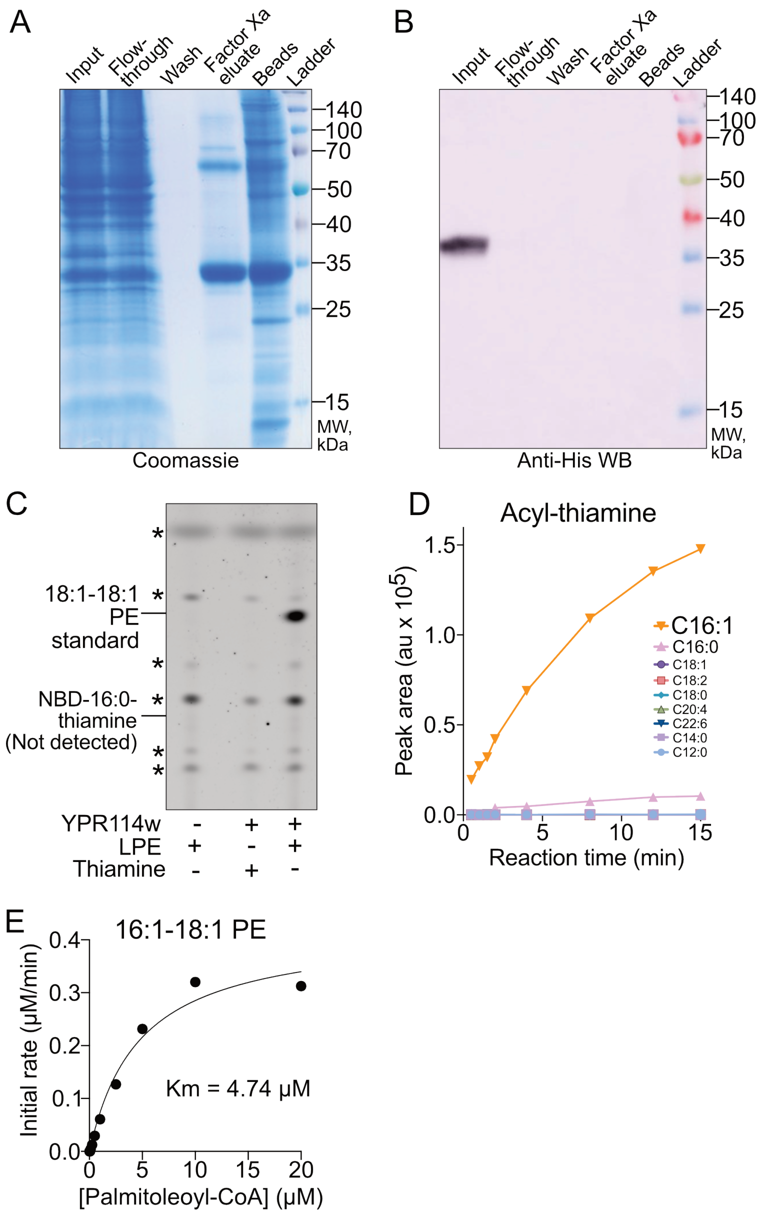

Fig. S14. Purification and enzymatic characterization of *S. cerevisiae* YPR114w protein.

A. Total protein Coomassie staining and (**B**) Western blot of the His tag in the indicated fractions obtained during 6xHis-Factor Xa site-tagged YPR114w purification using Ni-Sepharose. The protein was eluted via Factor Xa cleavage, removing the His tag from the eluate (termed 'Factor Xa eluate'), which was used in subsequent *in vitro* assays. **C.** Fluorescence imaging of thin-layer chromatography (TLC) separation of assays with NBD-palmitoyl-CoA, thiamine, and YPR114w. Non-specific bands in NBD-palmitoyl-CoA TLC are marked with '*'. Migration of NBD-palmitoyl-thiamine products and 18:1-18:1 PE standards are indicated. **D.** Time-dependent quantification of indicated acyl-thiamine species in reactions containing purified YPR114w, equimolar acyl-CoA mix and thiamine. **E.** Michaelis-Menten plot of initial reaction rates (V_0) in assays with purified YPR114w, 18:1-LPE, and increasing palmitoleoyl-CoA concentrations. All data shown are representative of two independent protein purifications. All MS/MS data were obtained in a targeted manner.

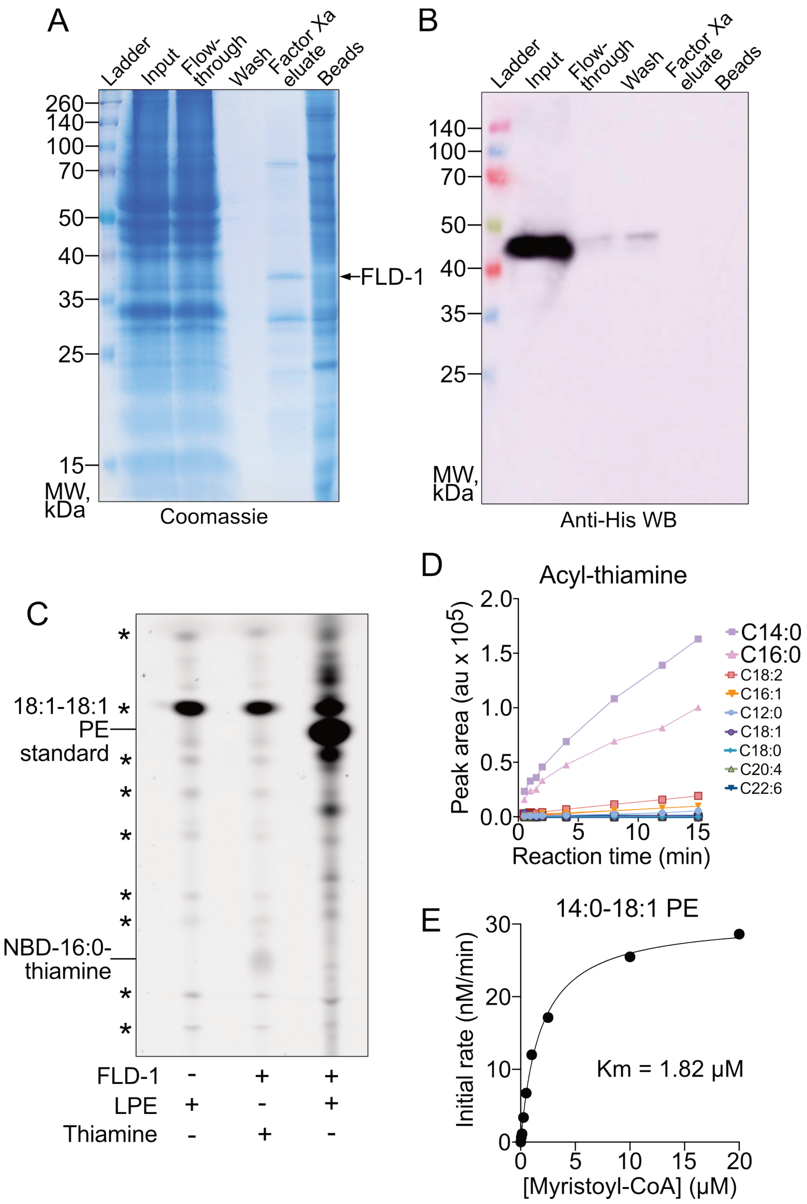

Fig. S15. Purification and enzymatic characterization of *C. elegans* FLD-1 protein.

A. Total protein Coomassie staining and (**B**) Western blot of the His tag in the indicated fractions obtained during 6xHis-Factor Xa site-tagged FLD-1 purification using Ni-Sepharose. The protein was eluted via Factor Xa cleavage, removing the His tag from the eluate (termed 'Factor Xa eluate'), which was used in subsequent *in vitro* assays. Band corresponding to the predicted molecular weight of FLD-1 is indicated. **C.** Fluorescence imaging of thin-layer chromatography (TLC) separation of assays with NBD-palmitoyl-CoA, thiamine, and FLD-1. Non-specific bands in NBD-palmitoyl-CoA TLC are marked with '*'. Migration of NBD-palmitoyl-thiamine products and 18:1-18:1 PE standards are indicated. **D.** Time-dependent quantification of indicated acyl-thiamine species in reactions containing purified FLD-1, equimolar acyl-CoA mix and thiamine. **E.** Michaelis-Menten plot of initial reaction rates (V_0) in assays with purified FLD-1, 18:1-LPE, and increasing myristoyl-CoA concentrations. All data shown are representative of two independent protein purifications. All MS/MS data were obtained in a targeted manner.

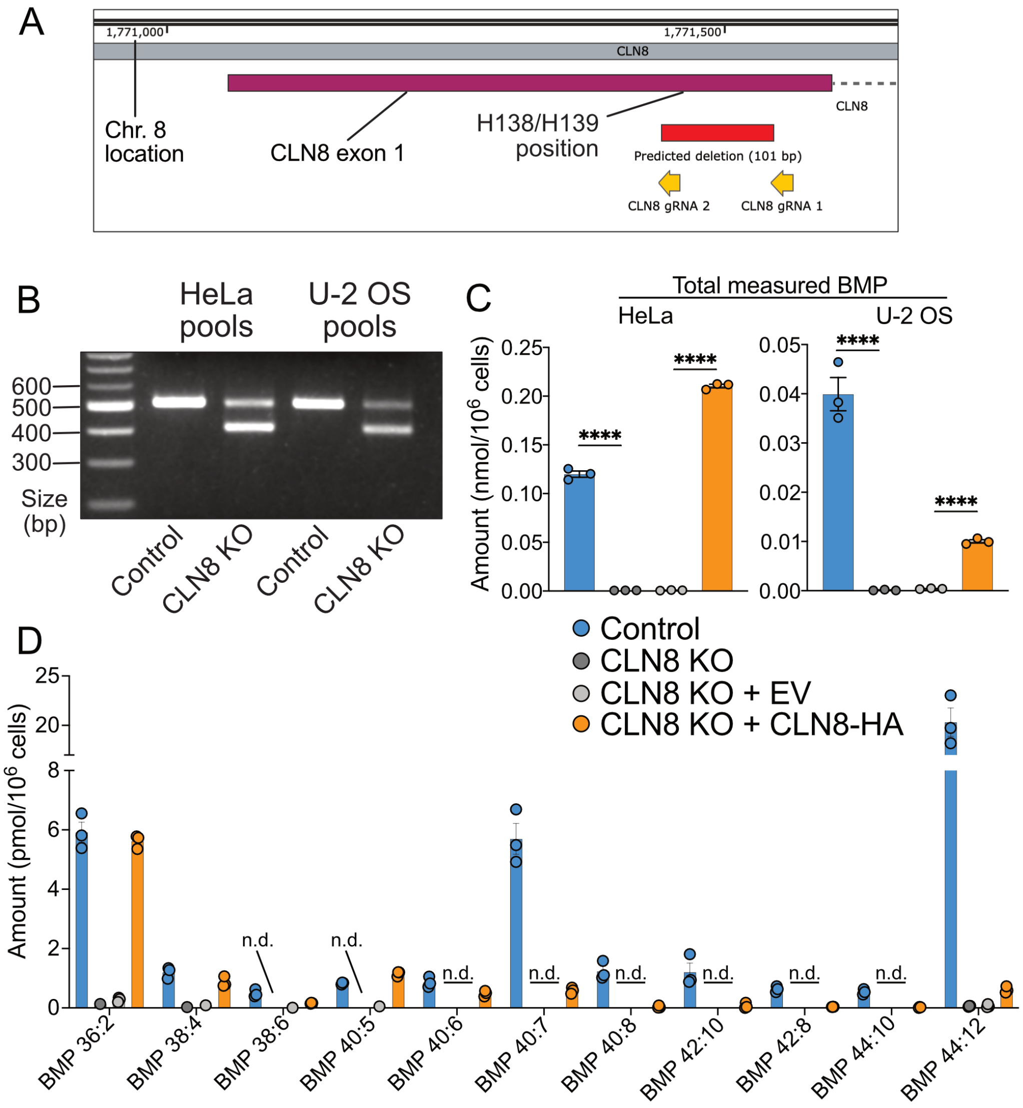

Fig. S16. Generation, validation and lipidomic characterization of CLN8 KO cell lines.

A. Schematic representation of CRISPR editing of the human CLN8 locus using a dual guideRNA excision strategy. **B.** PCR analysis of the CRISPR-targeted CLN8 locus in HeLa and U-2OS cells, comparing control and CLN8 knockout (KO) pools. The positions of the 1 kb DNA ladder bands are indicated on the left**.** Note that in our dual gRNA targeting strategy, each gRNA may independently induce a deletion of up to a few base pairs. However, the resulting CLN8 frameshift would yield a PCR band indistinguishable from the control, regardless of the presence a small deletion. **C.** The sum of all measured BMP species in HeLa and U-2OS control and CLN8 KO pools, transfected with either empty vector (EV) or CLN8-HA plasmid for 48 hours (n=3). **D.** The levels of indicated BMP species measured in U-2OS control and CLN8 KO pools, transfected with either EV or CLN8-HA plasmids (n=3). In C, ****- p< 0.0001 using one-way ANOVA with Dunnett's post-hoc test. N.d.- not detected. Data in (**C** and **D**) were obtained in an untargeted manner.

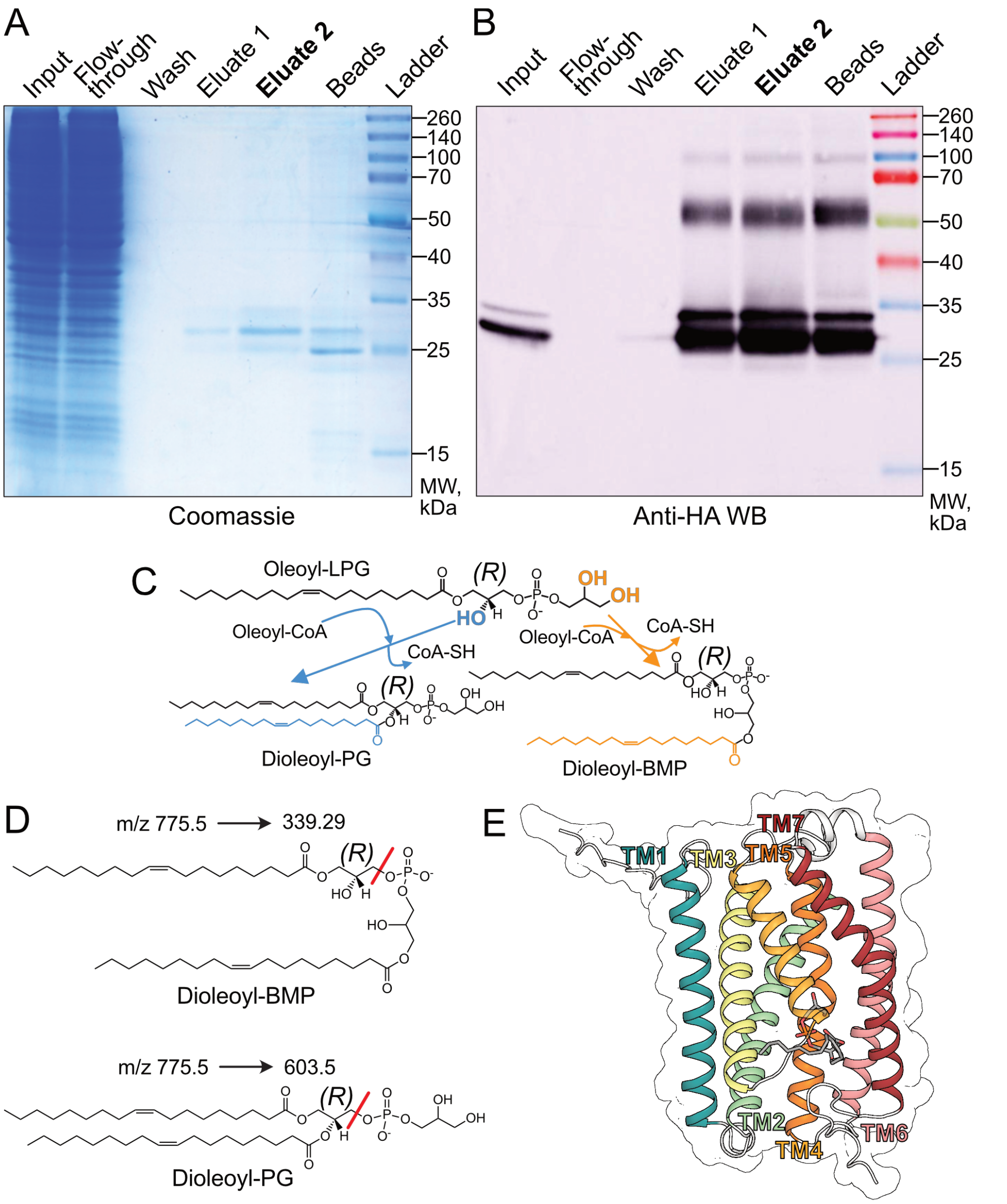

Fig. S17. Purification and enzymatic characterization of human CLN8 protein.

A. Total protein Coomassie staining and (**B**) Western blot of HA tag in the indicated fractions obtained during HA-tagged CLN8 purification using anti-HA magnetic beads. Eluate 1 represents the initial elution from beads using HA peptide, and eluate 2- size-exclusion column cleanup of purified CLN8. Eluate 2 was used in the *in vitro* assays. **C.** Schema showing potential acylation sites of 18:1-LPG, resulting in either 18:1-18:1-PG or 18:1-18:1-BMP formation. **D.** Schema showing the MS/MS fragmentation of PG and BMP. **E.** Molecular docking of 18:1-LPG to the AlphaFold2-predicted structure of CLN8, showing the protein from the side. Note that AlphaFold2 model confidence is very high (pLDDT > 90) and the predicted structure closely resembles the reported structure of the CLN8 paralogue CERS6 (*11*). **A** and **B** images are representative of two independent purifications.

Table S1. Details of antibodies used in this study.

**Target**	**Supplier**	**RRID**	**Catalogue No.**
FITC Anti-HA tag antibody	Abcam	AB_298835	ab1208
Monoclonal ANTI-FLAG® M2 antibody (mouse)	Sigma	AB_262044	F1804
HA Tag Polyclonal Antibody (SG77) (rabbit)	ThermoFisher	AB_2533988	71-5500
β-Tubulin (9F3) Rabbit mAb	Cell Signaling Technology	AB_823664	2128
HRP Anti-6X His tag® antibody	Abcam	AB_298652	ab1187
Recombinant Alexa Fluor® 647 Anti-GM130 (Golgi marker)	Abcam	AB_2889278	ab195303
anti-mouse IgG-HRP	Cell Signaling Technology	AB_330924	7076-S
anti-rabbit IgG-HRP	Cell Signaling Technology	AB_2099233	7074-S

Table S2. Details of chemicals and reagents used in this study.

Chemical	Supplier	Catalogue No.	CAS No.
18:1 lyso PA	Avanti	857130	325465-93-8
18:1 lyso PC	Avanti	845875	19420-56-5
18:1 lyso PE	Avanti	846725	89576-29-4
2-18:1 lyso PE	Avanti	855725	60701-97-5
18:1 lyso PG	Avanti	858125	326495-24-3
18:1 lyso PI	Avanti	850100	1246298-13-4
18:1 lyso PS	Avanti	858143	326589-90-6
18:1-18:1-PE	Avanti	850725	4004-05-1
18:1-18:1-PG	Avanti	840475	67254-28-8
18:1-18:1-BMP (*R,S*)	Avanti	857133	799268-67-0
Lauroyl-CoA lithium salt	Sigma	L2659	190063-12-8
Myristoyl-CoA lithium salt	Sigma	M4414	187100-75-0
Palmitoleoyl-CoA lithium salt	Sigma	P6775	18198-76-0
Palmitoyl-CoA lithium salt	Sigma	P9716	188174-64-3
Stearoyl-CoA lithium salt	Sigma	S0802	193402-48-01
Oleoyl-CoA lithium salt	Sigma	O1012	188824-37-5
Linoleoyl-CoA ammonium salt	Avanti	870736P	1246304-39-1
Arachidonoyl-CoA lithium salt	Sigma	A2056	188174-63-2
22:6-CoA ammonium salt	Avanti	870728P	800377-20-2
16-NBD-16:0 Coenzyme A	Avanti	810705	1367862-09-6
Oleoyl [1-^{14}C) Coenzyme A	American Radiochemicals	ARC0527	
Thiamine hydrocholoride	Sigma	102658221	67-03-8
Thiamine-d3 hydrochloride	Santa Cruz	sc-220232	
Palmitic acid (U-$^{13}C_{16}$, 98%)	Cambridge Isotope Labs	CLM-409-PK	56599-85-0
Stearic acid (U-$^{13}C_{18}$, 98%)	Cambridge Isotope Labs	CLM-6990-0.25	287100-83-8
Oleic acid (U-$^{13}C_{18}$, 98%)	Cambridge Isotope Labs	CLM-460-PK	287100-82-7
Bovine serum albumin (BSA)	Sigma	A8806	
FuGENE® HD Transfection Reagent	Promega	E2311	
Butanol	Sigma	33065	71-36-3
Methanol	Fisher Scientific	M/4056/17	67-56-1
Acetic acid	Sigma	695092	64-19-7

Chloroform	Fisher Scientific	C/4966/17	67-66-3
Heptane	Sigma	246654	142-82-5
Ethyl Acetate	Sigma	319902	141-78-6
SPLASH® LIPIDOMIX® Mass Spec Standard	Avanti	330707	
TLC silica gel 60, 200 x 200 mm, 250 µm, 60 Å plates	Merck	1.11845.0001	
InstantBlue® Coomassie Protein Stain	Abcam	Ab119211	
Spectra™ Multicolor Broad Range Protein Ladder	ThermoFisher	26634	
PVDF Transfer Membranes, 0.45 µm	ThermoFisher	88518	
Biotin	Sigma	B4501	58-85-5
Factor Xa protease	NEB	P8010L	
Ni-Sepharose™ excel resin	Cytiva	17371202	
Strep-Tactin®XT 4Flow® high capacity resin	Iba	2-5030-010	
Halt™ Protease Inhibitor Cocktail, EDTA-Free (100X)	ThermoFisher	1861278	
Pierce™ Anti-HA Magnetic Beads	ThermoFisher	88837	
SuperSignal™ West Pico PLUS Chemiluminescent Substrate	ThermoFisher	34580	
Pierce™ Lane Marker Reducing Sample Buffer	ThermoFisher	39000	
Lauryl Maltose Neopentyl Glycol (LMNG)	Anatrace	NG310	1257852-96-2
Decyl Maltose Neopentyl Glycol (DMNG)	Anatrace	NG322	1257852-99-5
n-Dodecyl β-maltoside (DDM)	Glycon Biochemicals	D97002	69227-93-6
Glyco-diosgenin (GDN)	Avanti	850525P	1402423-29-3

Table S3. Details of plasmids used in this study.

Plasmid name	Description	Source
3XMyc-EGFP-OMP25	Mito-IP control-MITO construct	Addgene #83355
3XHA-EGFP-OMP25	Mito-IP HA-MITO construct	Addgene #83356
pL_VSVG	CMV – VSVG (retrovirus packaging)	Gift from David Ron
pJK3	GAG-POL (retrovirus packaging)	Gift from David Ron
pCMV_TAT_HIV	Retrovirus packaging transactivator	Gift from David Ron
pcDNA5/FRT/TO	Backbone for Flp-In system used for protein purification	Thermofisher, V652020
pOG44	Flp recombinase	Thermofisher, V600520
pcDNA5/FRT/TO-TLCD1 WT	C-term FLAG/STREP tagged TLCD1 WT for Flp-In integration	This paper
pcDNA5/FRT/TO-TLCD1 D98A	C-term FLAG/STREP tagged TLCD1 D98A for Flp-In integration	This paper
pcDNA5/FRT/TO-TLCD1 H117A	C-term FLAG/STREP tagged TLCD1 H117A for Flp-In integration	This paper
pcDNA5/FRT/TO-TLCD1 H118A	C-term FLAG/STREP tagged TLCD1 H118A for Flp-In integration	This paper
pcDNA5/FRT/TO-TLCD1 E145A	C-term FLAG/STREP tagged TLCD1 E145A for Flp-In integration	This paper
pcDNA5/FRT/TO-TLCD1 E145K	C-term FLAG/STREP tagged TLCD1 E145K for Flp-In integration	This paper
pcDNA5/FRT/TO-TLCD1 R183A	C-term FLAG/STREP tagged TLCD1 R183A for Flp-In integration	This paper
pcDNA5/FRT/TO-TLCD1 R183E	C-term FLAG/STREP tagged TLCD1 R183E for Flp-In integration	This paper
pcDNA3.1(+)-C-HA	Backbone for transient cell expression studies	GenScript
pcDNA3.1-C-HA TLCD1 WT	C-term HA tagged TLCD1 WT for transient expression	This paper
pcDNA3.1-C-HA TLCD1 H54Y	C-term HA tagged TLCD1 H54Y for transient expression	This paper

pcDNA3.1-C-HA TLCD1 D98N	C-term HA tagged TLCD1 D98N for transient expression	This paper
pcDNA3.1-C-HA TLCD1 E145K	C-term HA tagged TLCD1 E145K for transient expression	This paper
pcDNA3.1-C-HA TLCD1 L225F	C-term HA tagged TLCD1 L225F for transient expression	This paper
pcDNA3.1-C-HA CLN8 WT	C-term HA tagged CLN8 WT for transient expression and purification	This paper
pYES2/CT	Backbone for yeast expression and purification	Thermofisher, V825120
pYES2/CT C-HIS-YPR114w	C-term Factor Xa site-8xHIS tagged YPR114w for yeast expression and purification	This paper
pYES2/CT C-HIS-FLD-1	C-term Factor Xa site-8xHIS tagged FLD-1 for yeast expression and purification	This paper

Table S4. Details of the targeted LC-MS/MS assays performed in this study. The transitions in bold were used for quantification. Where possible, transitions arising from unique daughter ions were selected. A second transition was also included for peak validation, as both transitions should exhibit identical retention times.

**Analyte**	**Parent ion (m/z)**	**Transition (m/z)**	**Cone voltage**	**Collision energy**
C12:0-thiamine	447.3	**122.07**, 126.04	50	50
C14:0-thiamine	475.3	**122.07**, 126.04	50	50
C16:0-thiamine	503.3	**122.07**, 126.04	50	50
C16:1-thiamine	501.3	**122.07**, 126.04	50	50
C18:0-thiamine	531.3	**122.07**, 126.04	50	50
C18:1-thiamine	529.3	**122.07**, 126.04	50	50
C18:2-thiamine	527.3	**122.07**, 126.04	50	50
C20:4-thiamine	551.3	**122.07**, 126.04	50	50
C22:6-thiamine	575.3	**122.07**, 126.04	50	50
12:0-18:1-PE	662.5	**521.5**, 603.5	50	20
14:0-18:1-PE	690.5	**549.5**, 603.5	50	20
16:0-18:1-PE	718.5	**577.5**, 603.5	50	20
16:1-18:1-PE	716.5	**575.5**, 603.5	50	20
18:0-18:1-PE	746.5	**605.5**, 603.5	50	20
18:1-18:1-PE	744.5	**603.5**	50	20
18:2-18:1-PE	742.5	**601.5**, 603.5	50	20
20:4-18:1-PE	766.5	**625.5**, 603.5	50	20
22:6-18:1-PE	790.5	**649.5**, 603.5	50	20
12:0-18:1-BMP	693.5	**247.3**, 339.3	50	20
14:0-18:1-BMP	721.5	**275.3**, 339.3	50	20
16:0-18:1-BMP	749.6	**313.3**, 339.3	50	20
16:1-18:1-BMP	747.6	**311.3**, 339.3	50	20
18:0-18:1-BMP	777.6	**341.3**, 339.3	50	20
18:1-18:1-BMP	775.6	**339.3**	50	20
18:2-18:1-BMP	773.6	**337.3**, 339.3	50	20
20:4-18:1-BMP	797.6	**361.3**, 339.3	50	20
22:6-18:1-BMP	821.6	**385.3**, 339.3	50	20
C16:0-d3-thiamine	506.3	**125.07**, 126.04	50	50
C18:0-d3-thiamine	534.3	**125.07**, 126.04	50	50
C18:1-d3-thiamine	532.3	**125.07**, 126.04	50	50
U-^{13}C16:0-thiamine	519.4	**122.07**, 126.04	50	50
U-^{13}C18:0-thiamine	549.4	**122.07**, 126.04	50	50
U-^{13}C18:1-thiamine	547.4	**122.07**, 126.04	50	50

Data S1. (separate file)

Raw peak areas, abundance in nmol, and molar composition of PE species measured in HeLa and U-2OS control and TLCD1 KO pools and clones (related to Fig. S3) in an untargeted manner.

Data S2. (separate file)

Raw peak areas, abundance in nmol, and molar composition of PC, PE and CL species measured in either whole cells or anti-HA immunoprecipitates of HeLa control and TLCD1 KO pools stably expressing either MYC- or HA-OMP25 protein (related to Fig. S4) in an untargeted manner.

Data S3. (separate file)

Raw peak areas, abundance in nmol, and molar composition of PC and PE species in wild-type, *ypr114w*Δ or *yjr116w*Δ *S. cerevisiae* strains (related to Fig. 3 and Fig. S13) in an untargeted manner.

Data S4. (separate file)

Raw peak areas and abundance in nmol of BMP species measured in HeLa and U-2OS control and CLN8 KO pools (related to Fig. 4 and Fig. S16) in an untargeted manner.

REFERENCES AND NOTES

1. M. Ruiz, R. Bodhicharla, E. Svensk, R. Devkota, K. Busayavalasa, H. Palmgren, M. Ståhlman, J. Boren, M. Pilon, Membrane fluidity is regulated by the C. elegans transmembrane protein FLD-1 and its human homologs TLCD1/2. *eLife* **7**, e40686 (2018).

2. A. Romanauska, A. Köhler, Lipid saturation controls nuclear envelope function. *Nat. Cell Biol.* **25**, 1290–1302 (2023).

3. W. J. Valentine, K. Yanagida, H. Kawana, N. Kono, N. N. Noda, J. Aoki, H. Shindou, Update and nomenclature proposal for mammalian lysophospholipid acyltransferases, which create membrane phospholipid diversity. *J. Biol. Chem.* **298**, 101470 (2022).

4. G. van Meer, A. I. P. M. de Kroon, Lipid map of the mammalian cell. *J. Cell Sci.* **124**, 5–8 (2011).

5. K. Petkevicius, H. Palmgren, M. S. Glover, A. Ahnmark, A.-C. Andréasson, K. Madeyski-Bengtson, H. Kawana, E. L. Allman, D. Kaper, M. Uhrbom, L. Andersson, L. Aasehaug, J. Forsström, S. Wallin, I. Ahlstedt, R. Leke, D. Karlsson, H. González-King, L. Löfgren, R. Nilsson, G. Pellegrini, N. Kono, J. Aoki, S. Hess, G. Sienski, M. Pilon, M. Bohlooly-Y, M. Maresca, X.-R. Peng, TLCD1 and TLCD2 regulate cellular phosphatidylethanolamine composition and promote the progression of non-alcoholic steatohepatitis. *Nat. Commun.* **13**, 6020 (2022).

6. E. Winter, C. P. Ponting, TRAM, LAG1 and CLN8: Members of a novel family of lipid-sensing domains? *Trends Biochem. Sci.* **27**, 381–383 (2002).

7. J. L. Kim, S. Ben-Dor, E. Rosenfeld-Gur, A. H. Futerman, A novel C-terminal DxRSDxE motif in ceramide synthases involved in dimer formation. *J. Biol. Chem.* **298**, 101517 (2022).

8. K. C. Whitfield, M. W. Bourassa, B. Adamolekun, G. Bergeron, L. Bettendorff, K. H. Brown, L. Cox, A. Fattal-Valevski, P. R. Fischer, E. L. Frank, L. Hiffler, L. M. Hlaing, M. E. Jefferds, H. Kapner, S. Kounnavong, M. P. S. Mousavi, D. E. Roth, M. Tsaloglou, F. Wieringa, G. F. Combs Jr., Thiamine deficiency disorders: Diagnosis, prevalence, and a roadmap for global control programs. *Ann. N. Y. Acad. Sci.* **1430**, 3–43 (2018).

9. Y. Zhao, Y.-Q. Chen, T. M. Bonacci, D. S. Bredt, S. Li, W. R. Bensch, D. E. Moller, M. Kowala, R. J. Konrad, G. Cao, Identification and characterization of a major liver lysophosphatidylcholine acyltransferase *. *J. Biol. Chem.* **283**, 8258–8265 (2008).

10. M. A. Zhukovsky, A. Filograna, A. Luini, D. Corda, C. Valente, The structure and function of acylglycerophosphate acyltransferase 4/ lysophosphatidic acid acyltransferase delta (AGPAT4/LPAATδ). *Front. Cell Dev. Biol.* **7**, 147 (2019).

11. T. C. Pascoa, A. C. W. Pike, C. S. Tautermann, G. Chi, M. Traub, A. Quigley, R. Chalk, S. Štefanic, S. Thamm, A. Pautsch, E. P. Carpenter, G. Schnapp, D. B. Sauer, Structural basis of the mechanism and inhibition of a human ceramide synthase. *Nat. Struct. Mol. Biol.*, 10.1038/s41594-024-01414-3 , (2024).

12. S. K. Mallela, R. Almeida, C. S. Ejsing, A. Conzelmann, Functions of ceramide synthase paralogs YPR114w and YJR116w of *Saccharomyces cerevisiae*. *PLOS ONE* **11**, e0145831 (2016).

13. A. I. P. M. de Kroon, Metabolism of phosphatidylcholine and its implications for lipid acyl chain composition in *Saccharomyces cerevisiae*. *Biochim. Biophys. Acta* **1771**, 343–352 (2007).

14. W. R. Riekhof, J. Wu, J. L. Jones, D. R. Voelker, Identification and characterization of the major lysophosphatidylethanolamine acyltransferase in *Saccharomyces cerevisiae*. *J. Biol. Chem.* **282**, 28344–28352 (2007).

15. M. Ruiz, R. Bodhicharla, M. Ståhlman, E. Svensk, K. Busayavalasa, H. Palmgren, H. Ruhanen, J. Boren, M. Pilon, Evolutionarily conserved long-chain Acyl-CoA synthetases regulate membrane composition and fluidity. *eLife* **8**, e47733 (2019).

16. S. Ranta, Y. Zhang, B. Ross, L. Lonka, E. Takkunen, A. Messer, J. Sharp, R. Wheeler, K. Kusumi, S. Mole, W. Liu, M. B. Soares, M. de Fatima Bonaldo, A. Hirvasniemi, A. de la Chapelle, T. C. Gilliam, A.-E. Lehesjoki, The neuronal ceroid lipofuscinoses in human EPMR and mnd mutant mice are associated with mutations in CLN8. *Nat. Genet.* **23**, 233–236 (1999).

17. A. di Ronza, L. Bajaj, J. Sharma, D. Sanagasetti, P. Lotfi, C. J. Adamski, J. Collette, M. Palmieri, A. Amawi, L. Popp, K. T. Chang, M. C. Meschini, H.-C. E. Leung, L. Segatori, A. Simonati, R. N. Sifers, F. M. Santorelli, M. Sardiello, CLN8 is an endoplasmic reticulum cargo receptor that regulates lysosome biogenesis. *Nat. Cell Biol.* **20**, 1370–1377 (2018).

18. L. Bajaj, J. Sharma, A. di Ronza, P. Zhang, A. Eblimit, R. Pal, D. Roman, J. R. Collette, C. Booth, K. T. Chang, R. N. Sifers, S. Y. Jung, J. M. Weimer, R. Chen, R. W. Schekman, M. Sardiello, A CLN6-CLN8 complex recruits lysosomal enzymes at the ER for Golgi transfer. *J. Clin. Invest.* **130**, 4118–4132 (2020).

19. J. Heravi, M. Waite, Transacylase formation of bis(monoacylglycerol)phosphate. *Biochim. Biophys. Acta* **1437**, 277–286 (1999).

20. M. Kousi, A.-E. Lehesjoki, S. E. Mole, Update of the mutation spectrum and clinical correlations of over 360 mutations in eight genes that underlie the neuronal ceroid lipofuscinoses. *Hum. Mutat.* **33**, 42–63 (2012).

21. S. Ranta, M. Topcu, S. Tegelberg, H. Tan, A. Üstübütün, I. Saatci, A. Dufke, H. Enders, K. Pohl, Y. Alembik, W. A. Mitchell, S. E. Mole, A.-E. Lehesjoki, Variant late infantile neuronal ceroid lipofuscinosis in a subset of Turkish patients is allelic to Northern epilepsy. *Hum. Mutat.* **23**, 300–305 (2004).

22. K. Reinhardt, M. Grapp, K. Schlachter, W. Brück, J. Gärtner, R. Steinfeld, Novel CLN8 mutations confirm the clinical and ethnic diversity of late infantile neuronal ceroid lipofuscinosis. *Clin. Genet.* **77**, 79–85 (2010).

23. V. K. Gowda, H. Vegda, K. Sugumar, G. Narayanappa, V. M. Srinivasan, R. Santhoshkumar, M. Bhat, S. Balu, M. R. Naveen, Neuronal ceroid lipofuscinosis: Clinical and laboratory profile in children from tertiary care centre in South India. *J. Pediatr. Genet.* **10**, 266–273 (2020).

24. Q. Zhang, D. Yao, B. Rao, L. Jian, Y. Chen, K. Hu, Y. Xia, S. Li, Y. Shen, A. Qin, J. Zhao, L. Zhou, M. Lei, X.-C. Jiang, Y. Cao, The structural basis for the phospholipid remodeling by lysophosphatidylcholine acyltransferase 3. *Nat. Commun.* **12**, 6869 (2021).

25. R. M. Robertson, J. Yao, S. Gajewski, G. Kumar, E. W. Martin, C. O. Rock, S. W. White, A two-helix motif positions the lysophosphatidic acid acyltransferase active site for catalysis within the membrane bilayer. *Nat. Struct. Mol. Biol.* **24**, 666–671 (2017).

26. S. Wang, M. Zhang, H. Sun, T. Li, J. Hao, M. Fang, J. Dong, H. Xu, Multi-omics analysis of TLCD1 as a promising biomarker in pan-cancer. *Front. Cell Dev. Biol.* **11**, 1305906 (2023).

27. U. N. Medoh, A. Hims, J. Y. Chen, A. Ghoochani, K. Nyame, W. Dong, M. Abu-Remaileh, The Batten disease gene product CLN5 is the lysosomal bis(monoacylglycero)phosphate synthase. *Science* **381**, 1182–1189 (2023).

28. T. Thornburg, C. Miller, T. Thuren, L. King, M. Waite, Glycerol reorientation during the conversion of phosphatidylglycerol to bis(monoacylglycerol)phosphate in macrophage-like RAW 264.7 cells. *J. Biol. Chem.* **266**, 6834–6840 (1991).

29. D. Bulfon, "Basic Insights into BMP Synthesis and its Role in Drug-Induced Phospholipidosis," thesis, Graz University of Technology, Graz, Austria (2019).

30. M. Waite, L. King, T. Thornburg, G. Osthoff, T. Y. Thuren, Metabolism of phosphatidylglycerol and bis(monoacylglycero)-phosphate in macrophage subcellular fractions. *J. Biol. Chem.* **265**, 21720–21726 (1990).

31. W. W. Chen, E. Freinkman, D. M. Sabatini, Rapid immunopurification of mitochondria for metabolite profiling and absolute quantification of matrix metabolites. *Nat. Protoc.* **12**, 2215–2231 (2017).

32. C. Janke, M. M. Magiera, N. Rathfelder, C. Taxis, S. Reber, H. Maekawa, A. Moreno-Borchart, G. Doenges, E. Schwob, E. Schiebel, M. Knop, A versatile toolbox for PCR-based tagging of yeast genes: New fluorescent proteins, more markers and promoter substitution cassettes. *Yeast* **21**, 947–962 (2004).

33. M. S. King, E. R. S. Kunji, Expression and purification of membrane proteins in *Saccharomyces cerevisiae*. *Methods Mol. Biol.* **2127**, 47–61 (2020).

34. Y. Tsuji, Transmembrane protein western blotting: Impact of sample preparation on detection of SLC11A2 (DMT1) and SLC40A1 (ferroportin). *PLOS ONE* **15**, e0235563 (2020).

35. D. Tegunov, P. Cramer, Real-time cryo-electron microscopy data preprocessing with Warp. *Nat. Methods* **16**, 1146–1152 (2019).

36. A. Punjani, J. L. Rubinstein, D. J. Fleet, M. A. Brubaker, cryoSPARC: Algorithms for rapid unsupervised cryo-EM structure determination. *Nat. Methods* **14**, 290–296 (2017).

37. L. Löfgren, G.-B. Forsberg, M. Ståhlman, The BUME method: A new rapid and simple chloroform-free method for total lipid extraction of animal tissue. *Sci. Rep.* **6**, 27688 (2016).

38. J. Eberhardt, D. Santos-Martins, A. F. Tillack, S. Forli, AutoDock Vina 1.2.0: New docking methods, expanded force field, and python bindings. *J. Chem. Inf. Model.* **61**, 3891–3898 (2021).

39. J. Jumper, R. Evans, A. Pritzel, T. Green, M. Figurnov, O. Ronneberger, K. Tunyasuvunakool, R. Bates, A. Žídek, A. Potapenko, A. Bridgland, C. Meyer, S. A. A. Kohl, A. J. Ballard, A. Cowie, B. Romera-Paredes, S. Nikolov, R. Jain, J. Adler, T. Back, S. Petersen, D. Reiman, E. Clancy, M. Zielinski, M. Steinegger, M. Pacholska, T. Berghammer, S. Bodenstein, D. Silver, O. Vinyals, A. W. Senior, K. Kavukcuoglu, P. Kohli, D. Hassabis, Highly accurate protein structure prediction with AlphaFold. *Nature* **596**, 583–589 (2021).

40. M. Varadi, D. Bertoni, P. Magana, U. Paramval, I. Pidruchna, M. Radhakrishnan, M. Tsenkov, S. Nair, M. Mirdita, J. Yeo, O. Kovalevskiy, K. Tunyasuvunakool, A. Laydon, A. Žídek, H. Tomlinson, D. Hariharan, J. Abrahamson, T. Green, J. Jumper, E. Birney, M. Steinegger, D. Hassabis, S. Velankar, AlphaFold Protein Structure Database in 2024: Providing structure coverage for over 214 million protein sequences. *Nucleic Acids Res.* **52**, D368–D375 (2024).

41. M. Varadi, S. Anyango, M. Deshpande, S. Nair, C. Natassia, G. Yordanova, D. Yuan, O. Stroe, G. Wood, A. Laydon, A. Žídek, T. Green, K. Tunyasuvunakool, S. Petersen, J. Jumper, E. Clancy, R. Green, A. Vora, M. Lutfi, M. Figurnov, A. Cowie, N. Hobbs, P. Kohli, G. Kleywegt, E. Birney, D. Hassabis, S. Velankar, AlphaFold Protein Structure Database:

Massively expanding the structural coverage of protein-sequence space with high-accuracy models. *Nucleic Acids Res.* **50**, D439–D444 (2022).

42. G. Bianchini, P. Sánchez-Baracaldo, TreeViewer: Flexible, modular software to visualise and manipulate phylogenetic trees. *Ecol. Evol.* **14**, e10873 (2024).

43. M. Sud, E. Fahy, D. Cotter, K. Azam, I. Vadivelu, C. Burant, A. Edison, O. Fiehn, R. Higashi, K. S. Nair, S. Sumner, S. Subramaniam, Metabolomics Workbench: An international repository for metabolomics data and metadata, metabolite standards, protocols, tutorials and training, and analysis tools. *Nucleic Acids Res.* **44**, D463–D470 (2016).

44. A. Schnellbächer, A. Zimmer, Stability and requirement for thiamin in a cell culture feed used to produce new biological entities. *Cells* **12**, 334 (2023).
